# Cross-Modal Musical Expectancy in Complex Sound Music: A Grounded Theory

**DOI:** 10.5334/joc.281

**Published:** 2023-07-04

**Authors:** Juan Pablo Correa

**Affiliations:** 1 Universidad Autónoma de Aguascalientes, Aguascalientes, MX

**Keywords:** Musical expectancy, Complex sound music, Cross-modality, Indexicality, Iconicity, Grounded cognition

## Abstract

Expectancy is a core mechanism for constructing affective and cognitive experiences of music. However, research on musical expectations has been largely founded upon the perception of tonal music. Therefore, it is still to be determined how this mechanism explains the cognition of sound-based acoustic and electroacoustic music, such as complex sound music (CSM). Additionally, the dominant methodologies have consisted of well-controlled experimental designs with low ecological validity that have overlooked the listening experience as described by the listeners.

This paper presents results concerning musical expectancy from a qualitative research project that investigated the listening experiences of 15 participants accustomed to CSM listening. Corbin and Strauss’ (2015) grounded theory was used to triangulate data from interviews along with musical analyses of the pieces chosen by the participants to describe their listening experiences.

Cross-modal musical expectancy (CMME) emerged from the data as a subcategory that explained prediction through the interaction of multimodal elements beyond just the acoustic properties of music. The results led to hypothesise that multimodal information coming from sounds, performance gestures, and indexical, iconic, and conceptual associations re-enact cross-modal schemata and episodic memories where real and imagined sounds, objects, actions, and narratives interrelate to give rise to CMME processes. This construct emphasises the effect of CSM’s subversive acoustic features and performance practices on the listening experience. Further, it reveals the multiplicity of factors involved in musical expectancy, such as cultural values, subjective musical and non-musical experiences, music structure, listening situation, and psychological mechanisms. Following these ideas, CMME is conceived as a grounded cognition process.

## Introduction

Expectancy is a default brain function through which individuals make affective and cognitive sense of their experiences by comparing the actual unfolding of events against their predictions (Nave et al., 2020). Through prediction, individuals make inferences, receive surprises, respond with adaptive behaviours, and build interactions in physical and social environments (Buckner & DiNicola, 2019; Yeshurun et al., 2021). In the arts, prediction leads to the comprehension of high-order structures and affective engagement (Brandman et al., 2021). Thus, expectancy is a core mechanism where affect and cognition converge to make sense of daily and aesthetic experiences.

In music listening, as an aesthetic experience, expectancy contributes to the comprehension of high-order formal structures (Williams et al., 2022), disambiguation of rhythmic, harmonic, and melodic functions (Leman & Maes, 2014; Tal et al., 2017), and induction of affects (Huron, 2006; Salimpoor & Zatorre, 2013). In summary, musical expectancy is a cornerstone of affective-cognitive musical phenomena.

However, most of the research on musical expectancy has employed tonal music. Few works have focused on non-metrical, sound-based music (Andean, 2010; Gatt, 2020; Roy, 1998), here conceptualised as complex sound music (CSM). As a result, whether the models developed with tonal music explain expectancy in CSM needs to be clarified.

Understanding expectancy in CSM is relevant because of several reasons. First, it is a subversive avant-garde genre constantly bringing about tradition-breaking sounds, instruments, instrumental techniques, and performance practices. Consequently, schematic predictions associated with tonal syntactic rules (Bharucha, 1994) are unlikely to have a role in CSM listening. Second, tradition-breaking practices might have caused marginalisation from the general audience (Landy, 1991) and cognition labs (Bailes & Dean, 2007; Mencke et al., 2022). Third, despite this marginalisation, a relatively small international community supports the creation of CSM. Hence, it is important to understand how the members of this community are affectively motivated by this marginalised music, particularly regarding the role of expectancy in the absence of syntactic rules. Fourth, CSM’s tradition-breaking sounds place this music in a perceptual midpoint between music and daily sonic experiences, making the results of this study relevant for both music cognition and general auditory perception.

Furthermore, the research on CSM expectations consists of theoretical frameworks (Andean, 2010; Gatt, 2020; Roy, 1998) and empirical studies relying on quantitative experimental designs (Bailes & Dean, 2007; Frey et al., 2009; Noble et al., 2020; Olsen et al., 2016). Those studies have not considered reflective descriptions of listeners’ experiences. Thus, they have not profited from the holistic approach offered by qualitative, naturalistic methods aimed at exploring the conditioning factors of the listening experience of music (Mencke et al., 2022).

Given the relevance of expectancy for the advancement of cognitive science, the lack of research on prediction in CSM listening, the importance of understanding how CSM enculturated listeners establish motivating listening experiences, and the dominant experiment-based approaches, this paper offers a qualitatively grounded framework that sheds light upon the conditioning factors involved in CSM expectancy in enculturated listeners, emphasising the role of cross-modality. Analyses of interviews and the musical pieces that were the object of reflection during the interviews were triangulated following Corbin and Strauss’ (2015) grounded theory. It is posited that this methodology is more appropriate to discover the complexity of music listening phenomena (Mencke et al., 2022; Schiavio et al., 2021) and to generate new meaningful hypotheses for barely investigated fields such as CSM expectancy.

I will argue that musical expectancy in CSM integrates multimodal information derived from (1) the unique acoustic features of CSM pieces; (2) the visually perceived actions and objects, as well as further acoustical information from the listening situation (e.g. performer’s unorthodox techniques, unusual instruments, and acoustic conditions of the listening space and technologies); and (3) indexical, iconic, and conceptual associations that arise from the listener’s past musical as well as non-musical sonic experiences. The integration of all or some of these sensory modalities (e.g., auditory, visual, proprioceptive, and interoceptive) into a single expectancy process fuels and re-enacts cross-modal schemata that enable prediction and induce affective-cognitive outcomes. Additionally, I will argue that CSM listening generates cross-modal expectations more readily and overtly than traditional music due to the subversive structure and practices that place CSM on a perceptual midpoint between music and daily sonic experiences.

## Complex Sound Music

I coined CSM after Schaeffer’s (1966) construct of *complex sound*: an ambiguous sonority that eludes classifications as either pitchless timbre, pitch, or harmony, and is composed by inharmonic spectra (i.e., perceptually close to the western category of noise). I defined CSM as a musical genre that comprises acoustic and electroacoustic repertoire characterised by inharmonic spectra; perceptually ambiguous sounds (between pitch and pitchless timbre); non-metrical rhythmic designs; and perceptually uncertain sound sources.

CSM belongs to a broader category of sonoristic music (Granat, 2009) that embraces timbre-driven works from Debussy onwards. Nevertheless, most CSM has been composed in the context of the post-1945 avant-gardes. Its radical subversion of traditional Western musical tenets has caused the diffusion of new values: expansions of the timbral limits of musical sound; an emphasis on duration, timbre, texture and intensity instead of meter, melody and harmony; a radical experimental stance; science and technology-based methods; and a continual creation of instruments, instrumental techniques and performance practices (Born, 1995; Gottschalk, 2016). I have argued elsewhere (Correa & Chamorro, 2023) that these values have been diffused by an international ‘sonoristic subculture’ that emerged from its Western classical music parent culture. The study participants are considered members of this subculture and are expected to share some of these values in the form of cultural schemata. Thus, it is assumed these values would condition musical expectancy.

## Musical Expectancy

Cultural schemata have been considered essential for musical expectancy since Meyer’s (1956) seminal work, although mainly regarding sound schemas of tonal styles. His hypothesis about the induction of affect when a ‘tendency to respond’ is inhibited (Meyer, 1956, p. 31) contains the core idea that a tendency to respond arises from ‘habit’. Meyer referred to habit responses as automatic behaviours developed by repetitive exposure to musical styles. I interpret this habit as part of the more general construct of ‘habitus’ defined by Bourdieu (2007) as a predominantly implicit, embodied pattern of practices an individual learns through social interactions. Therefore, musical expectancy is not only conditioned by mere exposure to music sound but by other practices expressed in, as well as conditioned by, the beliefs and behaviours of the members of subcultural groups and the affordances of physical (i.e., natural kinds) and social environments.

On the other hand, musical expectancy studies have contemplated two types of adaptive responses: affective (e.g., Steinbeis et al., 2006) and cognitive (e.g., Tillmann & Lebrun-Guillaud, 2006). In the former, we feel surprised, awed, tricked or disappointed when we listen to the realisation of a musical prediction (Huron, 2006). In the latter, a consequent musical event (i.e. a pitch or a chord) could give us a feeling of completion (e.g., when listening to the resolution of a dominant chord) or that something else must follow (e.g., after a tonic in second inversion, as typically happens right before the cadenza of a classical concerto). These feelings of closure and incompletion might intertwine affective responses with cognitive understandings. For example, we might understand musical form because we feel closures as resolutions or pending-to-resolve instabilities. Thus, affective and cognitive responses are integrated into a single phenomenon through the mechanism of expectancy. Furthermore, the results of this study suggest that this integration is facilitated by stimuli from different modalities coupled by associative and statistical learning. This coupling takes the form of indexical associations, which might be the foundations of more elaborate iconic and conceptual associations. These three constructs will be expanded on in the following sections.

In summary, most musical expectancy models (Bharucha, 1994; Huron, 2006; Jones, 1982; Margulis, 2005; Meyer, 1956, 1973) have mainly considered moment-to-moment predictions based on acoustic patterns and the effect of these predictions in two separated responses: affective and cognitive. Additionally, the influence of extramusical schemata such as subcultural values and behaviours (Fine & Kleinman, 1979), and the interrelation with other modalities that facilitate the affective-cognitive integration have yet to be noticed.

However, recent research on rhythm prediction has supported theories that involve cross-modal representations of rhythmic structure where sound and movement interrelate in sensorimotor simulations (Cheng et al., 2022; Gordon et al., 2018; Patel & Iversen, 2014). These studies and research on embodied musical cognition (see Maes, 2016) are relevant to the present work since their findings support the hypothesis that cross-modality is essential for musical expectancy.

## Cross-modality and Grounded Cognition

Cross-modality is at the core of grounded cognition theories. For example, Barsalou (2020) suggested that humans make sense of and learn from physical and social environments through a ‘Situated Action Cycle’. It starts with incoming cross-modal information from the environments (i.e. physical, social, and internal), which is assessed, consciously or pre-consciously, in terms of its self-relevance for the individual, and produces affective responses that influence actions and outcomes. The outcomes are partly by-products of prediction errors and restart the cycle by modifying our actions and perceptions in the environments. Every run of the cycle is stored in episodic and schematic memories to prepare us for future similar situations. In this way, new encounters with similar situations re-enact these memories through internal simulations, facilitating predictions and influencing our feelings and behaviours (Barsalou, 2009).

According to Barsalou (2020), re-enactment or simulation is fundamental for generating grounded meaning in linguistic interactions. However, Cayol and Nazir (2020) proposed that language-induced activity in modality-specific brain structures (LIAMBS) integrates information from different modalities to internally emulate the context associated with verbal concepts, serving us to anticipate and respond adaptively to that context but not necessarily to give meaning to the concepts. For them, meaning is essentially given by a linguistic system based on arbitrary grammatical rules that might sometimes use simulation and other mechanisms. Since CSM is not constructed upon arbitrary rule-based systems, as language and tonal music are, the LIAMBS framework supports the idea that simulation is the most suitable candidate to give sense to CSM listening experiences. I argue that musical-induced activity, in the form of re-enacted associative memories (i.e., indexical, iconic, and conceptual associations between sounds, objects, actions, and spaces) integrates affective-cognitive responses and induces cross-modal expectancy more readily and overtly in CSM than in tonal music.

## Grounded Cognition and Sound Indexes and Icons

Grounded cognition is vital to understand sounds as indexes and icons. Following Barsalou’s (2020) principles, it can be inferred that humans experience sounds embedded in physical and social contexts and associate them with their sources, producing actions, and environments. Humans store this information in episodic and schematic memories that integrate information from the different modalities of the situation (e.g., auditory, visual, and proprioceptive) and use these memories to anticipate adaptive behaviour in future sound experiences.

These associative memories give musical sounds the category of indexes, a sign that refers to its object because it is affected by it (Peirce, 1994). Accordingly, indexes are natural signs whose representative function ‘resides in and is ruled by objective reality’ (Sukhoverkhov, 2012, p. 156). Influenced by the results of the present study, I argue that associative and statistical learning allows us to build cross-modal schemata of the type sound-source-action (of production)-localisation-movement-space (SSALMS) to infer the features of the source (e.g., form, hardness, and size), actions of production (e.g., velocity, strength, and direction), localisations, and movements in space of any sound we perceive. We will infer real and imaginary sources, actions, and spaces (Young, 1996). The real will be a well-known source in a familiar situation (Siedenburg & Müllensiefen, 2019). The imaginary could range from relatively uncertain to very uncertain sources that we can imagine by re-enacting a relevant SSALMS. The inference mechanism is significant for acoustic CSM because its sonorities subvert traditional schemata, presenting us with novel timbres from unorthodox orchestration and instrumental techniques that weaken or efface previous instrumental ‘action-sound couplings’ (Jensenius, 2022). Furthermore, imaginary sources are crucial for electroacoustic music cognition since sounds are synthesised or deeply transformed and placed in composed virtual spaces through manipulations of intensity, reverberation, and virtual movement using stereophonic and surround-sound technologies (Normandeau, 2009).

On the other hand, memories also give musical sounds the category of icons. Drawing again from Peirce, an icon is a sign that refers to an object because it resembles its object. Musical sounds resemble other sounds, such as emotional voices (Coutinho & Dibben, 2013) and the sounds of nature (Pegg, 1992). Through this acoustic resemblance, they might indirectly resemble affective states, sizes, and shapes of animate and inanimate beings (Huron, 2015; Lakatos & McAdams, 1997). Musical sounds also resemble actions because, as natural kinds, they are the consequences (i.e., indexes) of actions (Handel, 1995). For example, a fast stroke produces a percussive attack (i.e., a sound that reaches its maximum intensity in a short time-lapse after its onset); thus, a percussive attack would resemble a particular fast action. Likewise, musical sounds resemble localisations, trajectories, and spaces because environmental sound sources move in space (Schroeder, 1970). Finally, musical sounds resemble materials, shapes and sizes because these are properties of natural sound sources (Giordano & McAdams, 2006). Following these ideas, these eight resemblances or iconic associations are built upon low-level indexical associations. Musical sounds resemble other sounds, actions, localisation, trajectories, spaces, materials, shapes and sizes because their sources, actions of production, localisations, and trajectories in space, whether real or imagined, resemble the objects of their iconic representations in any combination of these eight aspects.

## Methods

### Participants

There were fifteen participants aged between 20 and 56 (*M* = 44.4, *SD* = 12; 4 women, 11 men). Seven were professional acquaintances of the researcher, one responded to an invitation posted on a website devoted to contemporary music, three were directly invited by the researcher because of being acknowledged figures in the local contemporary art music scene, and the other four were recommended by other participants. The participants were chosen under the main criteria of their declared preferences for avant-garde music and their interest in contributing to the study. They reported a frequency of CSM listening that ranged from once a week to every day (*M* = 3.06, *SD* = 2.42).

The sample was intended to reach variation in terms of educational degree, musical training, years of listening to CSM (Min. = 2, Max. = 45, *M* = 25.2, *SD* = 13.14), nationality (7 Mexican, 3 Colombian, 3 Chilean, 1 Greek and 1 Korean) and country of residence (7 live in Mexico, 3 in Chile, 2 in Colombia, 2 in the USA, and 1 in France). Ten participants were professional musicians (4 PhD in Composition, 2 MA in Composition, 3 BA in music performance, and 1 BA in Music Education), one undergraduate music student, one philosophy teacher, one graphic designer, one administrative clerk, and one radio programmer. The philosophy teacher, the graphic designer, and the administrative clerk were the only participants with no musical training.

### Procedure

Once a potential participant responded to the invitation, the researcher organised a first encounter to explain the interview’s aims and to get information about CSM listening habits. The interview contents were explained to all participants as a description of their personal history with this kind of music and their experience with one or two CSM pieces of their choice. After each participant agreed, the piece was chosen, an informed consent containing demographical questions was signed, and the interview was scheduled.

Table 1 shows the pieces selected by the participants. Alphanumeric codes were used to protect participants’ identities. All interviews were realised and recorded online using Zoom. If the chosen piece was short (i.e., 3–10 minutes long), part of the interview consisted of listening to the entire piece and talking about the participant’s listening experience as the piece progressed. If it was long (i.e., more than 10 minutes), the dialogue focused on two or three relevant passages to the participant. Before the interviews, the researcher listened to the chosen pieces to get familiarised with their general features and form and be prepared to interact with the participants’ descriptions of their listening experiences. The interviews were transcribed for analysis.

**Table 1:** Pieces chosen by the participants to comment in the interviews.


PARTICIPANT	PIECE	COMPOSER	MEDIUM

P1	*Unbegrenzt*	Karlheinz Stockhausen	Unspecified instrumentation

P2	*Having Never Written a Note for Percussion*	James Tenney	Unspecified percussion

P3	*The Broken Harp*	Valeria Jonard	Comb and live electronics

P4	*Four Marys*	Julia Wolfe	String quartet

P5	*Formazioni*	Luciano Berio	Orchestra

P6	*Sinapsis*	Rodrigo Sigal	Guitar and tape

	*L’Orée du Compte*	Francis Dhomont	Acousmatic

P7	*Psychedelic*	Lucas Fagin	Septet with electronic instruments

P8	*Cymatics*	Panayiotis Kokoras	Amplified cymbal

P9	*Six Orchestral Pieces Op. 6, No. 1*	Anton Webern	Orchestra

P10	*Guero*	Helmut Lachenmann	Piano

P11	*Time, Motion and Memory*	John Young	Acousmatic

P12	*Como una Ola de Fuerza y Luz*	Luigi Nono	Piano, soprano, orchestra and tape

P13	*Guero*	Helmut Lachenmann	Piano

P14	*De Natura Sonoris No. 2*	Krzysztof Penderecki	Orchestra

P15	*Thallein*	Iannis Xenakis	Ensemble


### Data collection and analysis

The design followed a grounded theory approach (Corbin & Strauss, 2015). Data collection and analysis were performed through an iterative process. After each interview, preliminary results were obtained, allowing the researcher to refine strategies of analysis and collection for the upcoming interviews (i.e., to refine theoretical sampling). The cycle data collection-analysis-theoretical sampling was repeated until no new categories emerged and each category was defined according to the properties and dimensions that matched the complexity of each participant case (i.e., until achieving theoretical saturation).

Data was collected from two sources: the interviews and the musical analysis of the pieces. Ten of the 15 participants underwent two interviews; therefore, a total of 25 interviews were applied. Since the study aimed to understand the participants’ experiences, the first interview was unstructured (Seidman, 2006). This technique facilitated the researcher/interviewer’s stance of relative ignorance about the experience of the others (Spradley, 1979). Through this stance, the researcher tried to approach the experience as expressed by the participants and avoided exploring only pre-existent categories from the literature or the researcher’s experience. The essential interviewing techniques employed were listening actively to what participants had to say, trying not to interrupt them, and following up on what they said by asking and clarifying details about sequences of events, thoughts, images, feelings, and their history as avant-garde and CSM listeners. The main criteria to follow up on participants’ comments emerged with the iterative cycle data collection-analysis-theoretical sampling and consisted of understanding what has been determinant/relevant for the participants during their listening experiences, how their personal history with CSM listening has developed, and what kind of personal memories, interests, and features might have influenced their listening experiences. The purpose of the second interview was to clarify and validate the categories that emerged from the first interview as part of the iterative cycle mentioned above. Therefore, the second interview was mainly semi-structured; its guide consisted in the topics and questions that arose from the analysis of the first interview.

Concurrent with the analysis of the interviews, the musical pieces were analysed following an eclectic phenomenological methodology (Correa, 2020; Ferrara, 1984; Smalley, 1997). Since the object of the study was the listening experience, the object of the analysis was the sound. Even for the acoustic pieces, the object of analysis was the recorded versions referred to by the participants. Spectrograms and waveforms were obtained through the software iAnalyse as visual aids for the analysis. Scores were used mainly as references to clarify pitches, instrumentation, techniques, and measure numbers.

Data from the transcriptions of the interviews and musical analysis were triangulated in the iterative approach described above. Open coding was mainly implemented during the first interviews. Memos for codes were written from the first interview using Dedoose software. As the fieldwork progressed, axial coding dominated the analysis. In this stage, diagrams were used as visual aids to understand the dynamic interrelations of categories. Once theoretical saturation was achieved, the model was validated by mapping it through the 15 cases of the participants. Additionally, a brief presentation of the model and how it explained what was said in the interviews was sent to each participant to validate my interpretations.

## Results

**Figure 1:**
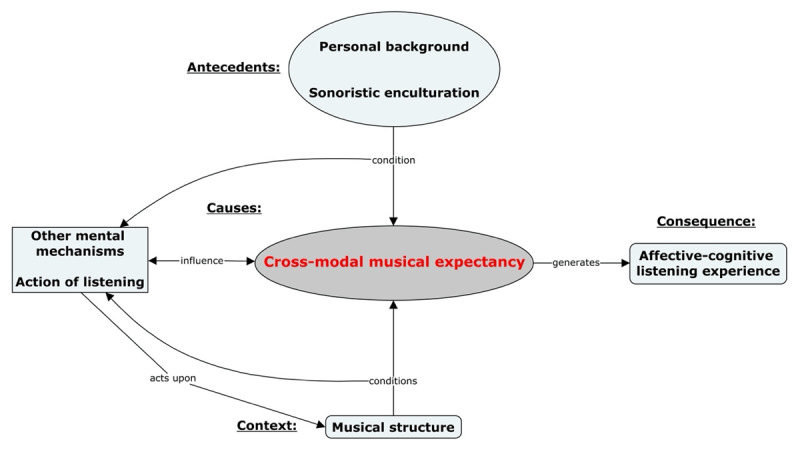
Diagram of the grounded model of affective-cognitive listening experience highlighting the mental mechanism of cross-modal musical expectancy as the paper’s main topic.

This paper presents the results concerning musical expectancy from a more comprehensive study that explored the listening experience of CSM-enculturated listeners. Cross-modal musical expectancy (CMME) emerged from the data as a subcategory of a nuclear phenomenon dubbed “affective-cognitive listening experience”. CMME was defined as a mental mechanism that processes the stimuli (i.e., the “musical structure”). Figure 1 is a diagram of a partial view of the more comprehensive model. Although the core phenomenon that emerged from the data was the “affective-cognitive listening experience”, CMME was drawn out of the other “mental mechanisms” and placed at the centre of the diagram, signalling it as the main topic of this paper.

Figure 1 shows the three conditioning factors for the listening experience: antecedents, causes, and context. Within this text, double quotation marks point at categories, properties, and properties’ dimensions of the grounded model when needed. Placed in the upper area of Figure 1 are two antecedent categories: “personal background” and “sonoristic enculturation”. They form an interrelated pair by providing cross-modal schemata and episodic memories that condition the “action of listening” and the “mental mechanisms”, expectancy included. In the lower area appears the context category: the “musical structure”. It comprises the acoustic properties of the pieces and other modalities afforded by the listening “situation” (e.g., performers’ actions). Since this context affords diverse formal dimensions, modalities, and saliency, it influences the “action of listening” that focuses on different events and dimensions of the musical structure and, in turn, activates appropriate “mental mechanisms”. Reciprocally, the mechanisms, which include expectancy, facilitate this focus. Since action of listening and mental mechanisms influence one another, they form another closely interrelated pair represented in the middle part of the diagram as the direct causes of the central phenomenon: the “affective-cognitive listening experience”.

In this grounded model, each of the main categories is defined in terms of their properties and the dimensions of the properties. For instance, one of the subcategories of the musical structure is “change”. It varies between two extremes: “infrequent-frequent” (see Table 2). On the other hand, dimensions can be types such as the types of “sonoristic values” and “acoustic features” shown in Table 2. Thus, properties and dimensions are subcategories that define the dynamism of the main categories.

**Table 2:** Main categories, properties and dimensions concerning the cross-modal musical expectancy process.


MAIN CATEGORIES	PROPERTIES	DIMENSIONS	PARTICIPANTS PER DIMENSION

Personal background	Personal memories	Episodic Schematic	1, 5, 8, 9, 10, 11, 13, 14 1, 2, 3, 4, 6, 8, 9, 10, 11, 12, 13, 14

	Personal features	Timbral sensitivity Curiosity	2, 3, 7, 8, 10, 11, 13 1, 3, 4,7, 8, 9, 10, 11, 13

	Personal interests	Musical Extramusical	2, 3, 6, 8, 9, 10 1, 8, 11

Sonoristic enculturation	Knowledge of the style	Superficial Deep	— 1, 2, 3, 5, 6, 7, 9, 10, 11

	Sonoristic values	Liking for timbre Innovation Radical stance	1, 2, 3, 4, 5, 6, 8, 9, 10, 11, 12, 13 1, 2, 3, 4, 5, 6, 7, 8, 9, 10, 11, 12, 13, 15 2, 7, 9, 10, 12, 13

Musical structure	Acoustic features	Spectrum/timbre Duration Texture Register Intensity	

	Change	Infrequent-Frequent Gradual-Sudden Unexpected-Expected	

	Formal dimension	Local-Global	

	Space	Composed-Real Static-Dynamic	

	Situation	Live Audio-visual Acousmatic	

Action of listening	Control	Low High	1, 2, 8, 10, 12, 13 1, 2, 3, 4, 5, 6, 7, 8, 9, 10, 11, 13, 14, 15

	Modality	Unimodal Cross-modal	1, 2, 3, 4, 5, 6, 7, 9, 10 1, 2, 3, 4, 6, 8, 10, 11, 13, 14, 15

	Formal focus	Local Global	1, 2, 3, 4, 5, 6, 7, 8, 9, 10, 13 1, 2, 4, 6, 7, 9, 10, 11, 12, 13, 14, 15

Mental mechanisms	Self-relevance appraisal	Idiosyncratic Cultural	1, 2, 3, 4, 5, 7, 8, 9, 10, 14, 15 2, 3, 7, 8, 10, 13, 15

	Attention	Covert Overt/Analytic	— 1, 2, 3, 4, 5, 6, 7, 8, 9, 10, 11, 13,

	Association	Indexical Iconic Conceptual	2, 3, 4, 5, 6, 8, 9, 10, 11, 13, 14 1, 3, 4, 5, 6, 8, 9, 10, 11, 12, 13, 14 1, 2, 3, 4, 5, 6, 7, 9, 10, 11, 12, 13, 15

	Musical expectancy	Unimodal Cross-modal	1, 3, 4, 5, 6, 7, 9, 10, 12, 13, 14 1, 3, 4, 6, 8, 10, 11, 13, 14

Affective-cognitive listening experience	Affects	Felt Recognised Negative Positive Non-specific Specific	1, 2, 3, 4, 5, 6, 7, 8, 9, 10, 11, 12, 13, 14, 15 5, 9, 11, 12, 13, 14 1, 2, 4, 5, 9, 10, 11, 12, 14 1, 2, 3, 4, 5, 6, 7, 8, 9, 10, 11, 12, 13, 14, 15 1, 2, 3, 4, 5, 6, 7, 8, 9, 10, 11, 12, 13, 14, 15 1, 4, 5, 14

	Concepts	Single Narrative	1, 3, 5, 6, 7, 8, 9, 10, 12, 13, 15 1, 2, 4, 6, 11, 13


Table 2 shows the hierarchical organisation of the six main categories, the properties, and the dimensions. The properties and dimensions in the Table were chosen according to their relevance to understanding the CMME process. These properties and dimensions will not be explicitly defined but implicitly explained in the cases presented in this section. Note that musical expectancy was included as a property of the mental mechanisms with its dimensions “unimodal and cross-modal”. The last column shows the numbers identifying the participants who expressed commentaries interpreted as the respective dimensions in the Table. The Appendix shows examples of these commentaries. Musical structure was excepted from this last column and the Appendix since data for this category mainly came from the musical analyses. Additionally, note that “covert attention” is a theoretical subcategory that could not be accessed through the interviews but represents the opposite extreme of “overt/analytical attention”. Participants explicitly reported the latter associated with a “highly controlled action of listening”.

The “personal background” was essential to enable CMME via a positive “self-relevance appraisal” of the stimuli. Its properties, “personal features”, through the dimensions “curiosity” and “timbral sensitivity”, and “personal interests” explained this appraisal and its affective consequences. For example, P10 described his first listening to *Guero* as a revelation. He described it as an assemblage of ‘completely undocumented’ sonorities that answered a personal search as a composer: ‘I was looking for sounds, but I couldn’t find what I actually wanted to hear, and I heard it with this piece’. P10 related that search to what he described as an innate curiosity connected to a particular sensitivity for timbre and his personal interests as a composition student.

“Personal memories” were also essential for CMME because they provided the substrate for “indexical, iconic, and conceptual associations”. For example, P10 described a particular interest in the initial gesture of *Guero* from the first listening, which was on a tape player. The initial gesture is a delicate rattle produced by rolling the fingers at different speeds and directions on the piano keyboard without drawing any pitches (see https://www.youtube.com/watch?v=sVHl-pqaIYM as a reference). P10 said: ‘I could see, even the first time I heard it, something was rolling […] I wasn’t sure how exactly he was producing the sounds […] it was rough […] it had some sort of regular edges.’ Later P10 said he could see the movement going to the left and the right at different speeds. He explained that this gesture evoked images of himself as a child producing this kind of rolling sound with ‘a stick against a fence’ and added: ‘I think, even unconsciously, these sounds are so primordial […] and kind of resonate with you very deeply’. That resonance means that ‘that sound energy […] engages [him] more, it makes [him] more related to this piece […] because [he has] more things to grasp.’

From these data, it could be inferred that P10 had a sound-source-action of production schema that activated an indexical association enabling him to imagine a generic shape and material density of the source and to predict the speed and direction of the sound-producing actions; that is, a CMME process within a “local formal dimension”.

Other examples of indexical associations involved in CMME in “acousmatic situations” (i.e., listening to music recordings) included P4’s failure to predict the spectromorphological possibilities of a string quartet: ‘I wanted to know how to get those timbres out of the instruments […] what a blow how they come to sound so different from the solo instrument’. Also, P11 constructed a narrative upon the affective-cognitive meanings of sound-source-action schemas: ‘The first part tells us about the beauty of this specific element that is the hinge […] but at the same time the composer knows that he cannot continue with that, and therefore the noises of running footsteps appear, which here break with that discourse, introducing an important dramatic element’.

On the other hand, some memories revealed iconic associations, such as P10’s resemblance between *Guero*’s rattling gesture and the rolling ‘stick against a fence’ from his childhood. These memories might have contributed to the expectancy experience as an episodic event and an instantiation of the indexical schema sound-source-action. Other examples of iconic associations were more abstract and contributed to the conceptual narrative side of CMME processes. Such was the case for P14’s apperception of specific moments of *De Natura Sonoris No. 2*: ‘It begins with a musical saw crying, singing. That first gesture really grows into that big moment […] that hammering gesture’ in measures 32 and 33, which she associated with the hammering of Christ’s crucifixion.

The common affective outcome of these CMME processes was an engaging, positive feeling. Nevertheless, there were other concurrent feelings such as ‘awe’ and ‘anxiety’ for P4, ‘scape’ and ‘drama’ for P11, ‘sadness’ and ‘tragedy’ for P14, and, for P10, what I interpreted as a positive appraisal of sound patterns that were not ‘just abstractions but […] part of [his] life’. This variation in the affective consequences was possibly due to the mental mechanisms interacting with the expectancy processes. The “non-specific” affect of engagement or motivation might have been a by-product of the self-relevance appraisal and the prediction process, while the more “specific” or categoric emotions were probably related to the content of the subjective iconic and conceptual associations.

To conclude with the illustration of CMME based on P10’s case, I propose that his CMME process received “overt attention” caused by a positive “self-relevance appraisal” of the “music structure”. The appraisal was due to what I interpret from the interviews as “liking for timbre”, “curiosity”, search for “innovation”, and “personal interest” in musical sounds that relate to his daily extramusical sonic experience. This appraisal facilitated the focus on a “local formal dimension” (i.e., the rattling gesture) and the sounds, objects, and actions interrelated in re-enacted cross-modal schematic and episodic memories that were essential to making sense of the musical passage. Therefore, it can be assumed that musical structure and associations were insufficient to start the CMME process. Instead, the self-relevance appraisal, conditioned by “personal features”, “personal interests”, and “sonoristic values”, triggered the process.

Other CMME cases involved online multimodal stimuli from “live” concert “situations”. For instance, P2 recalled his first experience with *Having Never Written a Note for Percussion* in a recital where the percussionist sat facing a tam-tam (see https://www.youtube.com/watch?v=Zd5WNTiwiU8 as a reference). The piece consisted of a continuous tremolando. It started in a nearly inaudible pianissimo, grew to a fortissimo, and returned to the first extremely low intensity. He read the programme notes and was prepared to hear nothing but a tremolando on a tam-tam. P2 said he was ‘marvelled’ by not being able to recognise the sound of the tam-tam once the piece had started: ‘that sound threshold at the beginning of the piece where one is not sure if one is imagining that things are already sounding or they are sounding, and the same at its end’. The beginning and the end of the piece were P2’s favourite moments, and they have since produced a similar affective outcome after 17 years and tens of new hearings in different “situations”. He said that ‘there is always a strange moment where the noise of the world’ fusions with the beginning and the end of the piece and added: ‘not being certain about when the piece begins and ends is what captures my attention’.

It is inferred that P2 was affectively engaged with the performance because it violated a cross-modal sound-source-action-space schema, which was covertly re-enacted since the moment he read the programme notes. The elements of such schema were the sound whose source was momentarily uncertain; the real source (the tam-tam); the action of tremolando; and the “real space” (i.e., the concert hall) and its noises (‘the noise of the world’) that blended with the extremely low intensity at the beginning and the end of the piece. I propose that during that first experience, P2 was pre-attentively receiving multimodal information from the performance (i.e., “musical structure”), but he could not confirm his cross-modal predictions. Once he realised this, he felt ‘marvelled’ by those moments of complete uncertainty about what was sonically occurring.

Thus, this is also a “local” CMME process. Further, similar to P10, this process might have been only possible due to P2’s reported “timbral sensitivity”: ‘when I was a child I had a secret fascination […] the noise of electrical generators […] I could stay fascinated, listening to that buzz for long periods […] Sometimes I would pick up the phone until the tone went off, just because I wanted to hear the beep’. I interpret that this timbral sensitivity, added to other sonoristic values, determined the positive “self-relevance appraisal” necessary to start the CMME process.

Other examples of being motivated by uncertainty or violations of sound-source-action-space schemata in “live” situations were observed in the cases of P3 and P6. They reported cross-modal schemata violations in live electroacoustic concerts because the instruments and the performers’ actions were not coherent with the sounds from the speakers. The incoherence was due to the effect of the electronics on the timbre and the movement of the sounds in the “composed space” (i.e., the virtual space created by electroacoustic techniques). P6 said: ‘[…] you are not sure if the resonance is coming from the guitar or the computer; that’s what I like.’ Later he added: ‘it stops the possibility of receiving confirmation of what you hear from what you see […] you hear the guitar being played in the centre [of the “real space”] and the [processed guitar sounds] go around the public as if the guitar were embracing it.’ In the case of P3, the first violation was the rarity of the instrument: a comb. She said: ‘knowing where the sound comes from causes curiosity or interest […] it is like an illusion [that] can only happen with a visual element, be it live or multimedia’. She also referred to the stereophonic movements of the electroacoustically transformed comb’s sounds as a kind of uncertain dynamism that maintained her engagement; she emphasised that had the space been “static”, it would have been ‘predictable’ and ‘not interesting’.

The presented cases showed an action of listening focused on “local” dimensions. Other cases like P13’s had a more “global formal focus” and produced what I interpret as a “narrative, conceptual association”. P13 also chose *Guero*. During his interview, we watched the YouTube version cited above. The piece starts with the commented rattling gesture on the white keys, which develops with some other pitchless plucks on the keys, strokes on the piano rim, resonances caused by depressing the sustain pedal, and, later, more rattling sounds on the black keys. At 2:03, the performance introduces resonant pitches from a gentle stroke on the piano’s harp and progresses by juxtaposing all these gestures in what P13 called a ‘subtle development’. From the musical analysis, the harp stroke at 2:03 is defined as a highly “unexpected change”, and from the interview data, it emerges as a positive predictive error that marks a reference point in P13’s narrative. The musical analysis also reveals “gradual, infrequent” timbral “changes” from non-resonating pitchless timbres to a mixture of resonating pitchless and pitched timbres. These timbres are drawn first from the outer parts of the piano (white keys and rim); then from the black keys that are spatially closer to the harp; and finally, from the harp.

I inferred that P13 built a narrative based on both the gradual timbral changes and the performers’ actions that progress from the outer to the inner parts of the piano. For P13, the piece represented the construction of a new instrument based on a metaphorical güiro, represented by the initial rattling, güiro-like gesture. From the interview data, I interpret P13 judged this gesture as ‘perplexing’ because it did not correspond with his cultural concept of the piano. I propose that perplexity was crucial for P13’s understanding of the gradual timbral changes as a construction of a new instrument because the rattling gesture, being an “unexpected” pianist’s performing action and an icon of the güiro, was a good candidate to symbolically start a gradual construction that evolves from a non-pianistic gesture to more recognisable piano sonorities. Thus, in this analysis, the performer’s actions represented a symbolic progression towards the final section (after 2:03). P13 identified this final section with the morphological ‘core’ of the piano (the harp) and the ‘heart of the piece’. He called it ‘the heart of the piece’ because he recognised that all the previous musical gestures juxtaposed in a ‘subtle development’. Consequently, I interpret this final section as the formal goal of P13’s narrative about constructing a new instrument, symbolising the fusion of the metaphorical güiro and the piano.

It can also be inferred that the main surprises he perceived (i.e., the ‘perplexing’ rattling gesture and the harp stroke on 2:03) were violations of cross-modal schemas that interrelate his cultural concept of the piano, the acoustical tendencies of the piece, and the performer’s actions he was watching (the YouTube video was his first encounter with the piece). Particularly, the harp stroke at 2:03 violated three tendencies: the pitchless timbral tendency; the tendency of the performer’s actions to roll the fingers on, and strike, the outer parts of the piano; and the conceptual tendency to regard the initial part of the performance as distant from the cultural/sonic schema of the instrument (i.e., a sound-source-action schema embedded in a cultural understanding). In conclusion, this was a CMME process across the entire piece that was realised through the use of indexical, iconic, and conceptual associations in interrelation with the spectromorphological tendencies of the piece.

The presented cases covered all the categories involved in CMME. “Personal memories” that fed “indexical, iconic, and conceptual associations”; “sonoristic values” as sources of cultural schemata that make participants enjoy, give meaning to, and expect complexity and uncertainty; “personal features” that together with the sonoristic values were essential for the “self-relevance appraisal” of CSM affordances; properties of the “musical structure” that constituted these affordances, triggered the mechanism of “expectancy”, and influenced the focus of the “action of listening”; and finally, the “affective-cognitive listening experience” that comprised both “non-specific” and “specific” affects such as liking, engagement, wonder, sadness and anxiety, and also “concepts” and “narratives” built upon, and contributing to, the expectancy processes. These findings point out the complexity of CMME in terms of its multiple factors and what traditional musical expectancy models are yet to address.

On the other hand, some of the experiences presented here could have been explained solely in terms of these traditional models (i.e. unimodally). For instance, P10’s prediction of the gesture’s speed can be explained by the gestalt law of good continuation in sound patterns; the surprise P13 received at 2:03 (which P10 also reported) can be interpreted as a result of a dynamic prediction (Huron, 2006); and other global processes described by P11, P13, and P14 can be explained as chains of predictions based on pure spectromorphological growth (Smalley, 1997). Furthermore, as seen in Table 2, not all participants reported CMME processes. Similarly, various cases of unimodal expectancy were observed. Thus, expectancy models based on the effect of acoustic features alone are pertinent to explain the findings of this study. Nevertheless, these models cannot account for the whole story.

## Discussion

This paper started by questioning whether the models of musical expectancy based on the perception of traditional music explain the cognition of CSM. Some results, particularly those not presented here (see unimodal expectancy processes in Table 2 and Appendix), can be explained solely by the more comprehensive models, e.g., Huron’s (2006) ITPRA. Nevertheless, I argue that these models have overlooked the cross-modal dimension of musical expectancy and, by doing so, the role of variables like the listening situation, extramusical episodic and schematic memories, and self-relevance appraisal. The listening situation, influenced by the “composed space” in live electroacoustic music and non-orthodox techniques in “live and audio-visual situations”, is especially relevant for CSM. That was illustrated in the cases of P2, P3, P6 and P13, supporting the general argument that ‘richer, multimodal experiences […] add layers of meaning and aesthetic affordance to the musical sound’ (Wald-Fuhrmann et al., 2021, p. 7). Also, extramusical environmental sonic experiences are particularly relevant for CSM since this music can be perceptually situated halfway between environmental sounds and music sounds. For instance, P10, P11 and P14 responded to the sounds by re-enacting movements of specific speed, acceleration, and spatial direction through what I interpret as indexical and iconic associations. These re-enactments might use similar neural mechanisms to those involved in the sensorimotor simulations proposed by Patel and Inversen (2014), Gordon et al. (2018), and Cheng et al. (2022), although in a non-metrical context. Finally, the effect of self-relevance appraisal was inferred in association with sonoristic values, personal features, and personal interests (e.g., P10’s curiosity and preference for sonorities that relate to his daily sonic experience and the sonoristic values shared by most participants, particularly liking for timbre (see Table 2 and Appendix)). Thus, personal antecedents and sonoristic values were necessary for the participants to attend to and construct CMME processes.

A general hypothesis derived from these results is that CMME processes initiate with the listener’s self-relevance appraisal of CSM’s affordances. This appraisal facilitates the interrelation of multimodal information derived from the listening situation and indexical, iconic, and conceptual associations with real and imagined sound sources, sound-producing actions, trajectories, and spaces. The sources of these associations are previous musical and non-musical sonic experiences encoded as episodic and schematic memories.

The category of indexical association plays a vital role in this CMME hypothesis. It encompasses the cross-modal nature of music listening and supports the argument that music structure transcends auditory stimuli and includes concomitant multimodal stimuli embedded in the listening situation. As Krueger (2009, p. 115) pointed out, ‘Musical experience always happens in context […] and the situated nature of music listening episodes plays a crucial role in shaping the character and content of [the subjective listening] experience’. Krueger’s situated nature of music listening is ecological and social; thus, it is multi and cross-modal. This was evident in the cases of P2, P3, P6, and P13, who relied upon the interrelation of visual information from the stage or the audio-visual, the “real space” acoustics, and the sounds coming from the performance. These sounds included the electroacoustic “composed space”. Table 2 shows that the “cross-modal action of listening” was observed in most of the participants. Therefore, to understand their listening experiences, it is necessary to conceptualise musical structure as a complex multi and cross-modal ecological phenomenon. These findings support the idea of an irreducible cross-modal situated nature of musical listening that makes music structure transcend the auditory modality and incorporate visual, proprioceptive and other sensory modalities depending on the situation.

The situated nature of listening encompasses all sonic experiences, not just music. This nature enables learning source-action-location-movement-space indexical associations to help make sense of other listening experiences. P10 expressed this type of association when he said he could see a ‘rolling’ action upon an object of ‘regular edges’. He did not refer to a specific object or action but to generic ones. P10’s case is analogous to Jensenius’ (2022, p. 102) example of generic action-sound couplings: ‘We do not […] see a guitar when hearing a guitar-like sound, but we get an embodied sensation of strings and a vibrating body’. This is the kind of indexical associations that constitutes what Godøy (2010) called ‘ecological knowledge’; a kind of knowledge that allows us to learn schematic and episodic cross-modal associations between situated sounds, sources, and spaces ‘in service of our orientation and survival’ (Godøy, 2003, p. 317).

Thus, what I call indexical association is equivalent to the essential mechanism that Godøy described as the foundation of musical affordances and meaning: the sound as ‘a transducer of *source-information*, meaning both the actions that go into producing the sound […], and the material properties of the sound source’ (Godøy, 2010, p. 106). Further, similar to Jensenius (2022), I propose that sound can also provide information about “real” and “composed” spaces and the trajectories of the sources in those spaces. Space can be perceived as real or imagined, and it could be congruent with terrestrial spaces or incongruent/fictional. That was part of the experiences described by P2, P3, and P6 when they failed to receive ‘confirmation of what [they heard] from what [they saw]’. I also propose that Godøy’s ecological knowledge is constituted by schemata that transcend instrumental sound-action couplings (Jensenius, 2022) and musical gestures that transduce into embodied meaning (Leman, 2010). Ecological knowledge encompasses sound-source-action-space associations from all sonic experiences. The results suggest that non-musical sonic experiences also condition music listening. Although, this might be more evident in the listening of CSM due to its subversive, environmental-like acoustical properties.

Related to the nature of these acoustical properties, Andean (2010) proposed a musical-narrative dichotomic model for acousmatic music compatible with the CMME hypothesis. The musical side of the dichotomic model is the formal structure perceived as pure sonic tendencies (i.e., “unimodal musical expectancy”), and the narrative side relies on our automatic predictions of sound sources (i.e., “indexical association”). Although Andean suggested that his framework only explained prediction in acousmatic repertoire that privileges easily identifiable sound sources, the results presented here suggest that Andean’s narrative side plays a similarly active role in CMME processes triggered by CSM electroacoustic and acoustic music that privileges more abstract sonorities.

Other theoretical (Gatt, 2020) and empirical (Frey et al., 2009; Olsen et al., 2016) works on CSM have focused on unimodal expectancy processes. The main difference between these works and the present study is how research questions were framed. These studies investigated how temporal changes in acoustic features influenced the perception/prediction of coherent phrases or groups in sound-based music. On the other hand, the present paper attempts to answer how idiosyncratic, cultural, and biological factors condition the perception of these temporal changes and the predictions we derive from them. While exploring this question, the data suggested these changes were not only afforded by the acoustic signal of the pieces but by the whole listening situation and the listeners’ personal and cultural history, as suggested by Krueger (2009) and Kozak (2020).

The impact of cultural values and personal features is an important contribution of the CMME hypothesis. Regarding sonoristic values, the participants generally expected innovative compositions, sometimes perceived as consequences of composers’ ‘radical postures’, as explicitly expressed by P2 and P7 (see Appendix). Additionally, the participants showed an educated listening for timbre (i.e., “liking for timbre”). Concerning personal features, many participants identified themselves as curious and possessing a “timbral sensitivity” traced back to an age before their sonoristic enculturation (see Table 2 and Appendix). In the model, these sonoristic values and personal features facilitated the CMME processes via self-appraisal.

Mencke et al. (2019) posed similar arguments. They maintained that personality traits like ‘openness to experience’ (related to “curiosity”) and cultural ‘frames’ (related to “knowledge of style” and “sonoristic values”) predispose listeners to enjoy and make sense of atonal music. Additionally, they contended that ‘pleasure from [atonal music] may arise from the rare recognition of regular structures and underlying patterns [because] such moments of increased regularity offer the opportunity to turn a weak into a strong predictive model’ (Mencke et al., 2019, p. 13). The results presented here do not contradict the latter hypothesis; nonetheless, they suggest that to test it, it might be more complex than expecting that through manipulating atonal music stimuli by inserting recognisable patterns in various degrees, ‘the more recognisable the pattern, the more the piece may be liked’ (Mencke et al., 2019, p. 13). The pleasure and motivation derived from listening to the uncertain in real situations, as presented in the cases of P2, P3, and P6, suggest that personal features, knowledge of the style and cultural values might mediate this liking effect leading to situations where the more recognisable the pattern, the less the piece may be liked.

That was quite clear when P2 said: ‘as the crescendo becomes more and more evident to me the piece is less interesting […] because it becomes incontrovertible that the performer is playing, and that she is playing a particular instrument’. Another example was P1: ‘in that part the qualities of the saxophone are [timbrally] obvious, […] I don’t like that […] and that’s when I grab onto something else, not the saxophone’. P1 was an example of how personal memories and knowledge of the style interfered with the self-relevance appraisal of the stimulus. I interpret that he decided to redirect his action of listening towards other sonic threads in the piece because the assessment of the saxophonist’s performance did neither match his aesthetic goals nor his predictions about timbre and style: ‘if the saxophonist is not playing special effects, it tends to remind you traditional music, I don’t like it’.

This view aligns with Barsalou’s (2020) ‘Situated Action Cycle’ that starts with a self-relevance appraisal of the perceived events regarding the individual’s believes, goals and cultural values to induce affects and move the individual to action. P1 was moved by the affective consequences of the negative appraisal to redirect his action of listening, adapting his behaviour to avoid displeasure and ensure more engaging listening. From this view, CMME is like the complex ‘Situated Action Cycle’ that integrates the diverse domains of grounded cognition (i.e. affect, perception, body structure and movement, physical environment, social interactions and cultural values) and moves the action of listening adaptively.

The CMME hypothesis is founded in a grounded cognition paradigm. The Situated Action Cycle helps unify the CMME framework with other cognitivist models of musical expectancy. CMME can be conceived as a cycle that involves the self-relevance appraisal of multimodal musical stimuli. The origin of the criteria of that appraisal could be idiosyncratic and cultural; thus, it shows an enormous variation (see Appendix). The appraisal itself is an expectancy process that initiates even before the listening experience, helping to prepare the individual for the listening event. Affective responses arise from the appraisal and move the action of listening towards, or away from, other prediction processes the stimulus/event will trigger. If the event is not rejected, it will re-enact cross-modal schemas and episodes whose sources include musical and non-musical sonic experiences. The prediction, as in other models, consists of what might happen and when it might be happening; thus, like Huron’s (2006) ITPRA model, attention and tension would be invested until the consequent event occurs. Then, new affective responses arise from successful predictions, prediction errors, and new self-relevance appraisals.

The CMME cycle complements or extends other music expectancy models, such as ITPRA, in three main aspects: first, it is sustained by self-relevance appraisal; second, the action of listening is focused on a multimodal structure determined by the listening situation and re-enacted memories; and third, these memories consist of cross-modal schemata and episodes derived from musical and non-musical sonic experiences. In the case of the ITPRA model, a self-relevance appraisal based on cultural values and idiosyncratic criteria is not considered, the event/stimulus consists of only musical sounds, and the schematic and episodic memories that lead to predictions are not conceived as re-enactments and come exclusively from auditory musical experiences. Hence, models like ITPRA partially explain a complex CMME process. In this complex process, musical expectancy cannot happen removed from other concomitant stimuli of different modalities, associative memories, and sensorimotor simulation brought about by these memories.

Furthermore, most previous musical expectancy models considered mainly pitch-based music. The perceptual closeness of CSM’s and everyday sounds had a decisive impact on the configuration of the CMME model. A relevant question arises from this argument: would the general CMME hypothesis be valid only for CSM? This study cannot answer the question, although ways can be suggested. While traditional music is based on strict, culture-driven syntactical systems, I propose CSM is based on ‘complex sounds’ (Murail, 2005) organised for aesthetic purposes, but perceptually localised halfway between musical and environmental sonorities. Therefore, a syntactical organisation in CSM must loosely follow general esthetical/cultural rules and natural/physical sonic rules. Hence, there might be at least two kinds of mechanisms to process musical sounds: one based on arbitrary or conventional musical systems and the other based on biological tendencies to use sound indexes; that is, to recognise sound sources, actions, locations, and trajectories in space (Agus et al., 2019; Hjortkjær et al., 2018; Kolarik et al., 2022).

This view is supported by Cayol and Nazir’s (2020) LIAMBS model, in which simulations influence but are not essential for understanding the meaning of words since meaning is given by the ‘linguistic system’. Traditional music conveys no semantic meaning, but as stated before, we understand the musical structure in terms of groups with resolving or pending-to-resolve closing gestures. To achieve this understanding, we use statistically derived metrical, melodic, and harmonic rules (Huron, 2006), equivalent to Cayol and Nazir’s rule-based linguistic system. Nonetheless, we might also use simulation systems to indexically and iconically re-enact how closing gestures are produced. For example, a dominant chord could resolve into a tonic by decreasing intensity and slowing down the pulse. The diminuendo and the rallentando might re-enact motor gestures of distended ending actions, facilitating the feeling of resolution, although solely the voice-leading might be sufficient to convey that feeling. The rule-based system of traditional music might have culturally overshadowed simulation systems because, historically, it has been the explicit focus of appreciation and creative procedures in Western cultures. On the contrary, CSM’s lack of a rule-based system constrains its cognition to simulation systems, making it more prone to induce overt CMME.

This constraint could have facilitated the emergence of the CMME cycle from the data as a sort of ITPRA model that includes self-relevance appraisal, the cross-modal nature of the stimuli, and the re-enactment of cross-modal episodic and schematic memories. The participants’ cross-modal experiences support this view and suggest that indexical associations are essential in this re-enactment. Despite indexes’ essential role, the evidence cannot fully support the theoretical assumption about low-level indexes as foundations of iconic and conceptual associations.

On the other hand, the results do not suggest that simulation systems are only relevant for CSM cognition. It has been argued that simulation is crucial for disambiguating our experience of traditional music (Cox, 2016; Leman & Maes, 2014; Tal et al., 2017). CMME is an area of research that needs more attention in both traditional and avant-garde music. The qualitative approach described here might be valuable for bringing attention to such research. This idea is supported by qualitative studies that have considered the relationship between personal, cultural, and musical factors in the construction of aesthetic experiences (Mencke et al., 2022); the impact of music on mental health and well-being (Bibb & McFerran, 2018); and the musical experience of performing (Geeves et al., 2016). Qualitative approaches, particularly those based on grounded theory, have the potential to generate ecologically valid hypotheses that reflect the complexity of rarely studied phenomena. In the present study, not being constrained by a set of pre-conceived hypotheses allowed the emergence of categories relevant to the phenomenon as the participants perceived it.

## Limitations

Relevant categories and the complexity of the phenomenon emerged from this qualitative analysis. However, as a grounded theory this study can only provide hypotheses to be tested through other methods. Furthermore, details of the cognitive process cannot be accessed. For instance, it is impossible to know if reported CMME started pre-consciously, or if cross-modality appeared once the process became accessible to consciousness. Similarly, it is not feasible to disentangle the order in which indexes, icons and concepts contributed to expectancy. Additionally, I suggested memories were crucial for CMME, but an alternative explanation based on Gibson’s (1966) ‘self-attunement’ to the invariants of the stimuli might be equally probable. Thus, other methods must be used to overcome such limitations.

Also, specific to this study is the fact that only three participants had no musical training (i.e., P11, P12, and P13). Although, there were no general differences observed between those three participants and the rest, in terms of the subcategories and their dimensions (see Table 2 and Appendix), exploring the experiences of more non-professional musicians could reveal such differences. Further, focusing on enculturated listeners to understand their positive experiences around CSM potentially renders the model less relevant for general audiences. Future empirical studies on CSM listening should include participants from different musical cultures and backgrounds to obtain a more comprehensive model that explains the effect of diverse enculturation processes and personality traits.

On the other hand, the assumed influence of CSM on the role of non-musical sonic memories in CMME makes it necessary to explore the CMME hypothesis in the experience of pitch-based music. It might be beneficial for the understanding of the listening experience to design studies that compare CSM and tonal music listening. For instance, if the acoustic properties of CSM make this music rely mainly upon simulations to induce CMME, then affective responses to CSM should be less intense in patients with environmental sound agnosia (Vignolo, 2003) than the responses of normal controls of equivalent cultural backgrounds and musical experience. On the contrary, both groups’ responses to tonal music should be similar.

Finally, not every participant described experiences interpreted as CMME (see Table 2 and Appendix). This does not necessarily mean they did not experience CMME. Other explanations could be found. For example, the interviewing method could not cover the participants’ whole range of listening experiences. This aligns with results suggesting that the listening experience depends on the listening situation’s affordances. Other explanations could be that most CMME processes might occur covertly and pre-consciously, and that showing overt CMME experiences might be prevented by subjective variables; the covert CMME and the subjective variables being out of the interviewing techniques’ reach. For these reasons, at this stage of the model, general similarities and differences between participants can only be interpreted as evidence of the dynamical existence of CMME’s conditioning factors, but not the extent to which the model explains the CSM-enculturated subgroups or individuals’ experiences. Hence, the model is expected to be generalisable to a population of CSM-enculturated listeners.

## Conclusion

This investigation of musical expectancy in CSM further reveals that musical listening is a multifaceted, complex experience. The results suggest that CSM structure extends beyond the auditory stimuli. The participants’ action of listening could not be disentangled from the situation it was embedded in nor from relevant re-enacted musical and extramusical past experiences. Barsalou’s situated action cycle framework helped to understand CMME as a complex process that incorporates cognitivist expectancy models’ principles and puts them in interaction with mechanisms like self-relevance appraisal and re-enacted associative memories grounded in the entire sonic experience of the listener, not just music listening. Due to the novel practices of CSM, sounds were significantly appreciated as indexes of sources, actions, and dynamic spaces, beyond mere continuations of low-level gestalt and high-level abstract sound patterns. Sound indexes emerged as key elements for CMME. They appeared as a construct that unifies a tridimensional structure of the listening experience founded on enculturation, subjective experiences, and mental mechanisms. Regarding the enculturation and the subjective experience, the participants might have learned to use sounds as indexes through their interactions within physical and social environments. Regarding the mental mechanisms, sound indexes, in the Peircean sense, have been part of all sound-sensitive species because sonic waves existed before the arrival of life on earth. Thus, sound indexes should be understood as an environmental source of natural selection pressure that enabled living things to acquire sound perception as a skill to guide effective actions and interactions in natural environments and allowed modern humans to develop music listening as one of the most distinctive features of their kind.

## Data Accessibility Statement

The data that support the findings of this study are available on request from the author. They are not publicly available due to information that could compromise the privacy of participants.

## Additional File

The additional file for this article can be found as follows:

10.5334/joc.281.s1 Appendix. Examples of excerpts, coded as the properties’ dimensions, according to the participants listed in the last column of Table 2.

## References

[1] Agus, T. R., Suied, C., & Pressnitzer, D. (2019). Timbre Recognition and Sound Source Identification. In K. Siedenburg, C. Saitis, S. McAdams, A. N. Popper & R. R. Fay (Eds.), Timbre: Acoustics, Perception, and Cognition (pp. 59–85). Springer International Publishing. DOI: 10.1007/978-3-030-14832-4_3

[2] Andean, J. (2010). The Musical–Narrative Dichotomy: Sweet Anticipation and some implications for acousmatic music. Organised Sound, 15(2), Article 2. DOI: 10.1017/S1355771810000099

[3] Bailes, F., & Dean, R. T. (2007). Facilitation and Coherence Between the Dynamic and Retrospective Perception of Segmentation in Computer-Generated Music. Empirical Musicology Review, 2(3), 74–80. DOI: 10.18061/1811/28854

[4] Barsalou, L. W. (2009). Simulation, situated conceptualization, and prediction. Philosophical Transactions of the Royal Society B: Biological Sciences, 364(1521), 1281–1289. DOI: 10.1098/rstb.2008.0319 PMC2666716 19528009

[5] Barsalou, L. W. (2020). Challenges and Opportunities for Grounding Cognition. Journal of Cognition, 3(1), Article 1. DOI: 10.5334/joc.116 PMC7528688 33043241

[6] Bharucha, J. J. (1994). Tonality and expectation. In R. Aiello & J. A. Sloboda (Eds.), Musical perceptions (pp. 213–239). Oxford University Press.

[7] Bibb, J., & McFerran, K. S. (2018). Musical recovery: The role of group singing in regaining healthy relationships with music to promote mental health recovery. Nordic Journal of Music Therapy, 27(3), 235–251. DOI: 10.1080/08098131.2018.1432676

[8] Born, G. (1995). Rationalizing Culture: IRCAM, Boulez, and the Institutionalization of the Musical Avant-Garde. University of California Press. https://www.degruyter.com/document/doi/10.1525/9780520916845/html. DOI: 10.1525/9780520916845

[9] Bourdieu, P. (2007). El sentido práctico. Siglo XXI.

[10] Brandman, T., Malach, R., & Simony, E. (2021). The surprising role of the default mode network in naturalistic perception. Communications Biology, 4(1), Article 1. DOI: 10.1038/s42003-020-01602-z PMC7815915 33469113

[11] Buckner, R. L., & DiNicola, L. M. (2019). The brain’s default network: Updated anatomy, physiology and evolving insights. Nature Reviews Neuroscience, 20(10), Article 10. DOI: 10.1038/s41583-019-0212-7 31492945

[12] Cayol, Z., & Nazir, T. (2020). Why Language Processing Recruits Modality Specific Brain Regions: It Is Not About Understanding Words, but About Modelling Situations. Journal of Cognition, 3(1), Article 1. DOI: 10.5334/joc.124 PMC7528693 33043245

[13] Cheng, T.-H. Z., Creel, S. C., & Iversen, J. R. (2022). How Do You Feel the Rhythm: Dynamic Motor-Auditory Interactions Are Involved in the Imagination of Hierarchical Timing. The Journal of Neuroscience, 42(3), 500–512. DOI: 10.1523/JNEUROSCI.1121-21.2021 34848500 PMC8802922

[14] Corbin, J., & Strauss, A. (2015). Basics of Qualitative Research: Techniques and Procedures for Developing Grounded Theory (4^a^, Kindle). SAGE Publications.

[15] Correa, J. P. (2020). El análisis del contenido emocional de la música y cómo usarlo para una ejecución expresiva (1^a^). UAA.

[16] Correa, J. P., & Chamorro, J. A. (2023). Música Sonorista: Definición de un Meta-género Desde una Perspectiva Antropológica. J. A. Chamorro, M. Coca & J. L. Rangel (Eds.), Miradas Multidisciplinarias en Torno al Arte y la Cultura. Universidad de Guadalajara.

[17] Coutinho, E., & Dibben, N. (2013). Psychoacoustic cues to emotion in speech prosody and music. Cognition & Emotion, 27(4), Article 4. DOI: 10.1080/02699931.2012.732559 23057507

[18] Cox, A. (2016). Music and Embodied Cognition: Listening, Moving, Feeling, and Thinking. Indiana University Press. DOI: 10.2307/j.ctt200610s

[19] Ferrara, L. (1984). Phenomenology as a Tool for Musical Analysis. The Musical Quarterly, 70(3), Article 3. DOI: 10.1093/mq/LXX.3.355

[20] Fine, G. A., & Kleinman, S. (1979). Rethinking Subculture: An Interactionist Analysis. American Journal of Sociology, 85(1), 1–20. DOI: 10.1086/226971

[21] Frey, A., Marie, C., Prod’Homme, L., Timsit-Berthier, M., Schön, D., & Besson, M. (2009). Temporal Semiotic Units as Minimal Meaningful Units in Music? An Electrophysiological Approach. Music Perception: An Interdisciplinary Journal, 26(3), Article 3. DOI: 10.1525/mp.2009.26.3.247

[22] Gatt, M. (2020). Memory, Expectation and the Temporal Flux of Acousmatic Music. Organised Sound, 25(2), 205–213. DOI: 10.1017/S1355771820000114

[23] Geeves, A. M., McIlwain, D. J., & Sutton, J. (2016). Seeing yellow: ‘Connection’ and routine in professional musicians’ experience of music performance. Psychology of Music, 44(2), 183–201. DOI: 10.1177/0305735614560841

[24] Gibson, J. J. (1966). The senses considered as perceptual systems. George Allen & Unwin LTD.

[25] Giordano, B. L., & McAdams, S. (2006). Material identification of real impact sounds: Effects of size variation in steel, glass, wood, and plexiglass plates. The Journal of the Acoustical Society of America, 119(2), 1171–1181. DOI: 10.1121/1.2149839 16521778

[26] Godøy, R. I. (2003). Motor-Mimetic Music Cognition. Leonardo, 36(4), 317–319. DOI: 10.1162/002409403322258781

[27] Godøy, R. I. (2010). Gestural Affordances of Musical Sound. In R. I. Godøy & M. Leman (Eds.), Musical Gestures (pp. 103–125). Routledge. DOI: 10.4324/9780203863411

[28] Gordon, C. L., Iacoboni, M., & Balasubramaniam, R. (2018). Multimodal Music Perception Engages Motor Prediction: A TMS Study. Frontiers in Neuroscience, 12. https://www.frontiersin.org/articles/10.3389/fnins.2018.00736. DOI: 10.3389/fnins.2018.00736 PMC6201045 30405332

[29] Gottschalk, J. (2016). Experimental Music Since 1970. Bloomsbury Publishing USA. DOI: 10.5040/9781501396328

[30] Granat, Z. (2009). Rediscovering “Sonoristics”: A Groundbreaking theory from the Margins of Musicology. In Z. Blažeković & B. D. Mackenzie (Eds.), Music’s Intellectual History (pp. 821–833).

[31] Handel, S. (1995). Timbre perception and auditory object identification. B. C. J. Moore (Ed.), Hearing (Vol. 2, pp. 425–461). Academic Press. DOI: 10.1016/B978-012505626-7/50014-5

[32] Hjortkjær, J., Kassuba, T., Madsen, K. H., Skov, M., & Siebner, H. R. (2018). Task-Modulated Cortical Representations of Natural Sound Source Categories. Cerebral Cortex, 28(1), 295–306. DOI: 10.1093/cercor/bhx263 29069292

[33] Huron, D. (2006). Sweet Anticipation: Music and the Psychology of Expectation (Kindle). MIT Press. DOI: 10.7551/mitpress/6575.001.0001

[34] Huron, D. (2015). Affect induction through musical sounds: An ethological perspective. Philosophical Transactions of the Royal Society of London. Series B, Biological Sciences, 370(1664), Article 1664. DOI: 10.1098/rstb.2014.0098 PMC4321139 25646521

[35] Jensenius, A. R. (2022). Sound Actions: Conceptualizing Musical Instruments. DOI: 10.7551/mitpress/14220.001.0001

[36] Jones, M. R. (1982). Music as a stimulus for psychological motion: Part II. An expectancy model. Psychomusicology: A Journal of Research in Music Cognition, 2(1), Article 1. DOI: 10.1037/h0094266

[37] Kolarik, A. J., Moore, B. C. J., Cirstea, S., Raman, R., Gopalakrishnan, S., & Pardhan, S. (2022). Partial visual loss disrupts the relationship between judged room size and sound source distance. Experimental Brain Research, 240(1), 81–96. DOI: 10.1007/s00221-021-06235-0 34623459 PMC8803715

[38] Kozak, M. (2020). Enacting Musical Time: The Bodily Experience of New Music. Oxford University Press. DOI: 10.1093/oso/9780190080204.001.0001

[39] Krueger, J. (2009). Enacting Musical Experience. Journal of Consciousness Studies, 16(2–3), 98–123.

[40] Lakatos, S., & McAdams, S. (1997). The representation of auditory source characteristics: Simple geometric form. Perception & Psychophysics, 59(8), 1180–1190. DOI: 10.3758/BF03214206 9401453

[41] Landy, L. (1991). What’s the Matter with Today’s Experimental Music?: Organized Sound Too Rarely Heard. Routledge. DOI: 10.4324/9780203066003

[42] Leman, M. (2010). Music, Gesture, and the Formation of Embodied Meaning. In Musical Gestures (pp. 126–153). Routledge.

[43] Leman, M., & Maes, P.-J. (2014). The Role of Embodiment in the Perception of Music. Empirical Musicology Review, 9(3–4), 236. DOI: 10.18061/emr.v9i3-4.4498

[44] Maes, P.-J. (2016). Sensorimotor Grounding of Musical Embodiment and the Role of Prediction: A Review. Frontiers in Psychology, 7. https://www.frontiersin.org/articles/10.3389/fpsyg.2016.00308. DOI: 10.3389/fpsyg.2016.00308 PMC4778011 26973587

[45] Margulis, E. H. (2005). A Model of Melodic Expectation. Music Perception: An Interdisciplinary Journal, 22(4), Article 4. DOI: 10.1525/mp.2005.22.4.663

[46] Mencke, I., Omigie, D., Wald-Fuhrmann, M., & Brattico, E. (2019). Atonal Music: Can Uncertainty Lead to Pleasure? Frontiers in Neuroscience, 12, 979. DOI: 10.3389/fnins.2018.00979 30670941 PMC6331456

[47] Mencke, I., Seibert, C., Brattico, E., & Wald-Fuhrmann, M. (2022). Comparing the aesthetic experience of classic–romantic and contemporary classical music: An interview study. Psychology of Music. DOI: 10.1177/03057356221091312

[48] Meyer, L. B. (1956). Emotion and meaning in music. University of Chicago Press.

[49] Meyer, L. B. (1973). Explaining Music: Essays and Explorations. University of California Press. DOI: 10.1525/9780520333109

[50] Murail, T. (2005). The Revolution of Complex Sounds. Contemporary Music Review, 24(2–3), Article 2–3. DOI: 10.1080/07494460500154780

[51] Nave, K., Deane, G., Miller, M., & Clark, A. (2020). Wilding the predictive brain. WIREs Cognitive Science, 11(6), e1542. DOI: 10.1002/wcs.1542 32902122

[52] Noble, J., Bonin, T., & McAdams, S. (2020). Experiences of Time and Timelessness in Electroacoustic Music. Organised Sound, 25(2), 232–247. DOI: 10.1017/S135577182000014X

[53] Normandeau, R. (2009). Timbre Spatialisation: The medium is the space. Organised Sound, 14(3), 277–285. DOI: 10.1017/S1355771809990094

[54] Olsen, K. N., Dean, R. T., & Leung, Y. (2016). What Constitutes a Phrase in Sound-Based Music? A Mixed-Methods Investigation of Perception and Acoustics. PLoS ONE, 11(12), Article 12. DOI: 10.1371/journal.pone.0167643 PMC5172564 27997625

[55] Patel, A. D., & Iversen, J. R. (2014). The Evolutionary Neuroscience of Musical Beat Perception: The Action Simulation for Auditory Prediction (ASAP) Hypothesis. Frontiers in Systems Neuroscience, 8. DOI: 10.3389/fnsys.2014.00057 24860439 PMC4026735

[56] Pegg, C. (1992). Mongolian Conceptualizations of Overtone Singing (xöömii). British Journal of Ethnomusicology, 1, 31–54. DOI: 10.1080/09681229208567199

[57] Peirce, C. S. (1994). The Collected Papers of Charles Sanders Peirce (Edición electrónica). Harvard University Press.

[58] Roy, S. (1998). Functional and Implicative Analysis of Ombres Blanches. Journal of New Music Research, 27(1/2), Article 1/2. DOI: 10.1080/09298219808570743

[59] Salimpoor, V. N., & Zatorre, R. J. (2013). Neural interactions that give rise to musical pleasure. Psychology of Aesthetics, Creativity, and the Arts, 7(1), Article 1. DOI: 10.1037/a0031819

[60] Schaeffer, P. (1966). Treatise on Musical Objects: An Essay Across Disciplines (C. North & J. Dack, Trans.). Univ of California Press.

[61] Schiavio, A., Maes, P.-J., & van der Schyff, D. (2021). The Dynamics of Musical Participation. Musicae Scientiae. DOI: 10.1177/1029864920988319 PMC9449429 36090466

[62] Schroeder, M. R. (1970). Digital Simulation of Sound Transmission in Reverberant Spaces. The Journal of the Acoustical Society of America, 47(2A), 424–431. DOI: 10.1121/1.1911541

[63] Seidman, I. (2006). Interviewing as Qualitative Research: A Guide for Researchers in Education and the Social Sciences (3^a^). Teachers College Press.

[64] Siedenburg, K., & Müllensiefen, D. (2019). Memory for Timbre. In K. Siedenburg, C. Saitis, S. McAdams, A. N. Popper & R. R. Fay (Eds.), Timbre: Acoustics, Perception, and Cognition (pp. 87–118). Springer International Publishing. DOI: 10.1007/978-3-030-14832-4_4

[65] Smalley, D. (1997). Spectromorphology: Explaining sound-shapes. Organised Sound, 2(2), Article 2. DOI: 10.1017/S1355771897009059

[66] Spradley, J. P. (1979). The ethnographic interview. Harcourt Brace Jovanovich, Inc.

[67] Steinbeis, N., Koelsch, S., & Sloboda, J. A. (2006). The role of harmonic expectancy violations in musical emotions: Evidence from subjective, physiological, and neural responses. Journal of Cognitive Neuroscience, 18(8), Article 8. DOI: 10.1162/jocn.2006.18.8.1380 16859422

[68] Sukhoverkhov, A. (2012). Natural Signs and the Origin of Language. Biosemiotics, 5(2), 153–159. DOI: 10.1007/s12304-011-9123-3

[69] Tal, I., Large, E. W., Rabinovitch, E., Wei, Y., Schroeder, C. E., Poeppel, D., & Zion Golumbic, E. (2017). Neural Entrainment to the Beat: The “Missing-Pulse” Phenomenon. The Journal of Neuroscience, 37(26), 6331–6341. DOI: 10.1523/JNEUROSCI.2500-16.2017 28559379 PMC5490067

[70] Tillmann, B., & Lebrun-Guillaud, G. (2006). Influence of tonal and temporal expectations on chord processing and on completion judgments of chord sequences. Psychological Research, 70(5), Article 5. DOI: 10.1007/s00426-005-0222-0 16177925

[71] Vignolo, L. A. (2003). Music Agnosia and Auditory Agnosia. Annals of the New York Academy of Sciences, 999(1), 50–57. DOI: 10.1196/annals.1284.005 14681117

[72] Wald-Fuhrmann, M., Egermann, H., Czepiel, A., O’Neill, K., Weining, C., Meier, D., Tschacher, W., Uhde, F., Toelle, J., & Tröndle, M. (2021). Music Listening in Classical Concerts: Theory, Literature Review, and Research Program. Frontiers in Psychology, 12. https://www.frontiersin.org/articles/10.3389/fpsyg.2021.638783. DOI: 10.3389/fpsyg.2021.638783 PMC8110713 33986708

[73] Williams, J. A., Margulis, E. H., Nastase, S. A., Chen, J., Hasson, U., Norman, K. A., & Baldassano, C. (2022). High-Order Areas and Auditory Cortex Both Represent the High-Level Event Structure of Music. Journal of Cognitive Neuroscience, 34(4), 699–714. DOI: 10.1162/jocn_a_01815 35015874 PMC9169871

[74] Yeshurun, Y., Nguyen, M., & Hasson, U. (2021). The default mode network: Where the idiosyncratic self meets the shared social world. Nature Reviews. Neuroscience, 22(3), 181–192. DOI: 10.1038/s41583-020-00420-w 33483717 PMC7959111

[75] Young, J. (1996). Imagining the Source: The Interplay of Realism and Abstraction in Electroacoustic Music. Contemporary Music Review, 15(1–2), 73–93. DOI: 10.1080/07494469608629690

