# Supplementary material for: Cross-Modal Musical Expectancy in Complex Sound Music: A Grounded Theory
Source: J Cogn. 2023 Jul 4;6(1):33. doi: 10.5334/joc.281 (PMC10327858; doi:10.5334/joc.281)
Supplement: Appendix. — Examples of excerpts, coded as the properties’ dimensions, according to the participants listed in the last column of Table 2. [file joc-6-1-281-s1.pdf]

Appendix

This table complements Table 2 of categories, properties, dimensions, and participants who expressed commentaries interpreted as the respective dimensions in the table. It includes excerpts that serve as evidence of the analysis presented in the paper and the coding process and illustrate the variety within the model.

Categories	Properties	Dimensions	Excerpts
Personal antecedents	Personal memories	Episodic	P1: […] voluntarily or involuntarily, sometimes images of free jazz performance come to mind, and I don't like that.<br>P5: [...] I mean thoughts that are not strictly musical, that unfold because of the sonorities and have to do with associations […] for example, with memories, with moods, moments that are not quite pleasant but that I am interested in keeping in my memory.<br>P8: [...] I participated with several theatre groups and I think one of the things I liked the most was that [...] there were improvisation sessions where we played and [the actors] moved or reacted according to the sound [...]<br>P9: [...] that day I was there, looking at the landscape, it was green below and blue above, the sky, there were very few clouds and I love to contemplate that type of landscape that apparently does not have much but if you start to see details It's enormous [...] there I find that, that passivity of Felman [...]<br>P10: I made these sounds as a kid, I put in my bicycle, with a clip, a piece of paper or sometimes a plastic to make this kind of sound [and] even a stick against a fence, you know, I've done that.<br>P11: [...] the composer draws our attention to the beauty of these sounds, but at the same time challenges our memory because we have these sounds in our brain at a certain moment, on certain occasions [...]<br>P13: The Cure released an album called The Top and at the beginning of one of those songs they inserted a sound [of the gear] of a clock and it sounded something like [Guero's initial gesture].<br>P14: I knew this piece before, but because I was writing my piece [about hammering] I could associate [De Natura Sonoris' hammering] idea with my piece. Because it was my interest and that was the sound world I wanted to create.
		Schematic	P1: The moment I see someone tense it's like I'm getting tense because of someone else, and this music [improvised CSM] is like, here I am and this is me and now, I mean, I owe no one, I fear no one or anything.<br>P2: [...] I never imagined that it would happen to me, that it would take me so long, despite having the gong in front of me, to recognise it timbrically.<br>P3: [...] all you hear are the teeth of the comb […]<br>P4: I want to see how this was written because it's just a quartet […] what a blow how they come to sound so different from the solo instrument.<br>P6: [...] you can't avoid thinking about the source of the sound that is being presented [... Dhomont] allows you to say: - From what part of the real world do these things come to the magical world? -<br>P8: [...] like when you run the pencil through the spiral notebook [...] there were several times during college that I found that many composers [used] that synthetic and granular sound [...]<br>P9: [...] I love listening to the sea [...] the waves for me are that, they seem to be the same but if you listen to it for two hours it will change, something is changing [...]

			P10: I could hear that there was something rolling, and it was rough, somehow, it had some sort of regular edges.<br>P11: [...] I think that the choice of [sounds] of footsteps and shortness of breath speaks to us of a certain escape [...]<br>P12: I didn't [expect this sonority with the piano] because I heard it with this work [...] I had heard some Stockhausen pianos, but this was different [...]<br>P13: [...] there are times when one has played around with things and sounds like this come out [...]<br>P14: I was thinking, in order to hammer a huge nail, it needs some time and force, and how many times physically the people could do real hammering, and what is the gap in between […]
	Personal features	Timbral sensitivity	P2: There are certain noises in the environment that caught my attention and I could even say that they caught my attention before the study of music caught my attention.<br>P3: […] sensation or liking for those elements of the sound [...] it was there before [my musical studies], but I didn't know how to manifest them or how to name them.<br>P7: [...] more than the appreciation of timbre or the notion of timbre, there is the notion of gesture and sound production through an intuitive way [during my teenage ...]<br>P8: Since I was a child I have been listening to things like Pink Floyd that my dad used to play or an opera where a singer made super high-pitched notes that I said: - Wow! Can that be done with the voice? - I feel that perhaps that interest in sounds began there.<br>P10: […] it was very much into the soundscape, the church bell plus all the cicadas, because there were these […] huge cypress trees around the church, and then the bell, and they have the birds and the cicadas in the summer. So, that's very much in my memory,<br>P11: [...] Many times I keep listening to those noises [from the street...] a bit randomly, but suddenly I find that this sounds beautiful.<br>P13: I lived all my childhood life until I was 11 years old in the countryside, in the silence of the countryside, therefore, my most memorable experience in the middle of the countryside is listening to the sounds of the birds, of the grass, all of that [...]
		Curiosity	P1: I think curiosity is something we [people who like CSM] have in common, being curious.<br>P3: Since I was a student when I was naive, I don't know, I imagined things that people were already doing or are now doing, for me it was an experience: -what would happen if the sound surrounds you?- for instance.<br>P4: [...] we use a lot of Brian Eno's Oblique Strategies that open up a world of possibilities in all of the arts, so [...] often it consisted of changing the way your instrument traditionally sounds [...]<br>P7: [...] not being a classically trained musician from [childhood] gave me that freedom or that need to approach instruments in a very intuitive way that was not necessarily regulated by conventional technical learning.<br>P8: [... from my childhood] people who did different things called my attention a lot [...] because I felt that they stood out in a certain aspect.<br>P9: I've found that the more complex music, or the one in which I can't predict what's going to happen, the more it appeals to me.<br>P10: I think it's a personality thing; I'm always curious, and I want to see what's behind and what's inside things.<br>P11: [...] you have to leave Wagner to get to Schönberg. And that sensibility allows it, that's why people who are more square, more into metal music, can't achieve this, or it costs them much more. [...] sometimes it is not easy because you do not know where you are going, it is like getting away from your comfort zone.

			P13: [...] my job consists precisely in [...] having a more open mind for certain things [...] I believe that in this aspect contemporary music is a game, it is a game to be able to open up to [uncertainty].
	Personal interests	Musical	P2: When I got involved with minimalism, I thought that what interested me were [...] the chord structures they used, the instrumentation, etc., but as time went by I realised that what really caught my attention was what happens to the sound in time.<br>P3: What interests me about sound goes hand in hand with space, how sound is projected in space, how it lives in space, because it is more experiential.<br>P6: [...] I always identified the technological part as the element that attracted me, not the timbre, but they are actually closely related.<br>P8: [...] the sounds that even make my chest vibrate so that one feels that the body even rumbles are sounds that I really, really like [...]<br>P9: [...] this music communicates a lot of emotion to me because I am aware of the historic moment, I am aware of what those guys faced when making music, it is part of what I value a lot and, therefore, when I hear this music I give it that historical importance.<br>P10: Music used to be connected to all the sounds. In ancient times, the shamans, who were the musicians of the time, would imitate nature […] I feel more connected with this old-style music […]
		Extramusical	P1: In my spare time I'm a poet, and so these pieces from *Aus den Sieben Tagen* sort of integrate poetry, music, indeterminacy, so that's what motivated me to choose it [for this interview].<br>P8: [...] I have seen several performances where there is absolutely no sound and the performer is simply moving his body [...] I really like to see how a person moves, what she is capable of doing with his body.<br>P11: [...] I am a designer [...] when I talk about language it is the way of using the elements to organize a speech [...] the way in which he uses electronic sound [...] as he orders it, it has its moment, it has its rhythm, it has its height, it has its ups and downs, and that is going to make a sound discourse.
Sonoristic enculturation	Knowledge of the style	Superficial	
		Deep	P1: Here I am willing to listen to the effects of each one of the instruments without paying attention to the fact they are instruments, but I have the conception of this music as: it is the sound, it is not an instrument.<br>P2: [Minimalist CSM] is not structured upon harmonic or rhythmic ideas […] it is based on time as a structuring element […] precisely because they are extracting a raw fragment of time in which things happen.<br>P3: In this piece there are no elements that distract you from anything other than texture.<br>P5: There are moments where there is a chordal sonority that has worked in other pieces, in Chorus and especially in the third movement in Sinfonia where there are many sonorities that he uses in [Formazioni].<br>P6: [...] this is one of the things I like most about Francis Dhomont because [...] he gives a spectral space to each of the formal functions [of the texture] and he keeps it for the [whole piece].<br>P7: [...] from that piece onwards Fagin's music is marked by the use of synthesizers, he has an increasingly refined his writing for synthesizers, being much more precise about the timbre combinations he is going to choose, [and] the use of the electric guitar as a kind of symbol, I know this explicitly because of the friendship I have with Lucas [...]<br>P9: This piece presented me with a complex problem of expectation because each event is different from one another. There is no classic question-and-answer logic [...] each event is different from the other, but at its own time is consistent with the previous one. There is a very delicate chain of acts and consequences and as it happens

			in a very short time you can go very deep into that process.<br>P10: I started to do electronic music courses […] with a French composer who was a professor at Paris Conservatoire […] he introduced me to spectral music and all this stuff, first hand because his friends were Tristan Murail and Gérrad Grisey.<br>P11: [...] typical electronic beginning, the layers that rise suddenly, a louder part that is interrupted, another layer that rises [...] the presentation of this element, which is repetitive and serves as a point of reference, which is an element that brings us to reality, an obviously recorded sound, from the concrete old school [...]
	Sonoristic values	Liking for timbre	P1: I started [listening to this music] at the university [...] it all started with Farben [...] and then we heard things by Berio and Ligeti, and that's when I said: the timbres are not constrained or predetermined by the instruments.<br>P2: The act of composition seems to become more a situation of changing the sound space in which one is, rather than writing specific notes for a specific instrument.<br>P3: [...] to realise that sound is a physical phenomenon I think it was through the vision of two [composition] teachers that I had.<br>P4: [...] I had a group [of electronic music] with people from the university [...] from then on the synthesis of timbres has become very interesting to me.<br>P5: [In the conservatoire] around the age of 18 I heard a recording of something like a contest for contemporary composers of electronic music [...] effects achieved by means of voice distortion, by the distortion of the instruments, and the distortion of a text; all that was a timbral richness that caught my attention, and I think it is not an element that has ever been alien to me from then until now.<br>P6: The timbre is not something that appears if you don't pay attention [...] through what my dad always told me: - pay attention to it, listen - it's like [my interest in timbre] arose.<br>P8: Frank Zappa, through Frank Zappa I got to know Edgar Varèse's Ionisation [...] Frank Zappa was one of the [composers] who brought me closer to that music [of timbres].<br>P9: [...] there is another world in Sciarrino, there is the world of timbre [...] it is also contemplative [...] I enjoy that elegance [of his timbral design], but I look at it more from the outside, that is, I'm not so emotionally involved.<br>P10: I started to do electronic music courses […] with a French composer who was a professor at Paris Conservatoire […] he introduced me to spectral music and all this stuff, first hand because his friends were Tristan Murail and Gérrad Grisey.<br>P11: [...] I get the impression that these works were done with headphones because there are many subtleties that cannot be heard on speakers […] the guy rescues the beauty of those noises, of those specific sounds, of that lock […]<br>P12: [...] go looking for recordings in the street markets and there we used to find [...] these cassettes of contemporary music, electronic, electroacoustic, concrete music. At that moment we didn't know we were listening, but we liked it [...]<br>P13: [...] that is inside, so to speak, of the aesthetic politics of Stockhausen, of the madness of sounds, of timbres [...] in such a way that it is part of contemporary music [...]
		Innovation	P1: [The beauty in this music] lies in the spontaneity, in the randomness, in that it is never the same.<br>P2: One has to give up the very idea of what music is for one, otherwise it turns into auditory hell.<br>P3: [The space] had interested me because since the high school and undergraduate pieces there were several in

			which the instrument was on one side, or outside the room, or behind the audience, it was already a way of manipulating the sound in the space.<br>P4: [...] one really realized how to use the instrument in more adventurous ways, so with the two of them I delved into getting timbres out of [the instruments].<br>P5: Another thing that I like about this piece is the change in the traditional formation of the orchestra, [...] that helps to make the effect of the brass and woodwind more impressive […]<br>P6: […] my dad was very keen on experimental music and I listened to Stockhausen with him, and to progressive rock groups like Tangerine Dream.<br>P7: [...] In this piece, Lucas is not based on the archetypes of […] our generation [...] and on the other hand he tries to take from other musical languages that interest him, in particular the progressive rock of the 70s [...]<br>P8: [...] it's that pleasure of starting to find something different in music, one suddenly listens to rock bands and says: - Yes, there's a drum set, and a bass, there's a person singing and so on, but the guitar is doing something strange, he's doing something that sounds a little more strident, something louder -<br>P9: P9: [I appreciate this music because] they run into a new sound world. This music, Webern's first works, is precisely the music before he began to be contaminated by dodecaphonism [...] this music, like many others from this period, was considering how to communicate a musical idea without using schemes.<br>P10: […] he used the piano in a completely undocumented way for me, like I wouldn't think of it at that point.<br>P11: [...] I love Bach but you also have to have some courage and face the adventure of looking for other things.<br>P12: [...] rock had certain limits [...] when I started listening to the musicians of the [classical] tradition I realised that this doesn't end there [...] For example, The Rite of Spring [...] is impressive, but then my friend said to me - hey, I have the Pierrot Lunaire- Wow!, then you say - Wow!, all progress [classical music] is achieving -<br>P13: [...] but we are at the piano, but it is not the piano, clearly the sounds are not from the piano [...] the final experience that remains for me, once I hear all this, is that thing of experiencing the piano from a different perspective [...]<br>P15: I don't know why we humans like repetition a lot, that's why perhaps many times we don't listen to the new, but we prefer to recreate ourselves and insist on the auditory pleasures we receive from the music of the past.
		Experimentation	P2: Noise loses more and more space because even what was previously called noise I can eventually conceptualise in ways very similar to how I could conceptualise sounds of a musical instrument.<br>P3: [...] with the other composition teacher from Columbia we did many experiments; how to make a speaker and make things by hand.<br>P4: So we joined the experimental ensemble with [my trio] after a concert P2 conducted at the university.<br>P5: The composition workshop consisted of concerts where we presented our work played by colleagues or by ourselves, and on those occasions we could also listen to very interesting music, a little more experimental but very interesting.<br>P7: [... The pitch] was always a constraint for me, I always explored the gestures a little further […] Then there are musical marking encounters, in particular with the music of Iannis Xenakis; It was a music that had a considerable impact on my listening [...]<br>P8: [...] suddenly they make noises, the typical sounds of distortion, or that they make a hole in the tube amp, or they break the tube of the amp and it starts to sound something very shrill [...] it's that pleasure of starting to find something different in music [...]

			P9: [...] this period [free atonalism], personally seems to me the period of greatest musical exploration in the modern history of music [...]<br>P10: I was looking for interesting sounds; but at that point I didn't know how to do that [in my compositions …] Then, I heard that piece [Guero], and it what something else, you know, like, you don't[have to] hit the timpani anymore with the mallet, you can just use your fingernails.<br>P12: Going back [to the concert] by John Cage, [...] as a spectator I was perplexed because it is impressive, because you no longer played the piano, you just put your elbow on it, you touched the strings from behind [...]
		Radical stance	P2: [James Tenney] is a person closely linked […] with the rise of minimalism […] what you see is an impressive pedagogical clarity around the process.<br>P7: [...] what has always been interesting for me in the composition of music are the radical and well-assumed postures [... in the legend of eer] of Iannis Xenakis, without worrying that it was noise, texture, or whatever, his maximum concern was to produce an extreme experience of the perception in which he is playing all the time with the limit of what one can endure as a listener.<br>P9: [...] I feel more emotion in the link with the period of Webern and Schönberg because they underwent a radical change of language [...]<br>P10: So he really limited the sound palette to a very weird selection of sounds. Almost to must not sounding sounds, […] the least interesting sounds you would pick […] So he stuck to that and he was very precise with that selection, the sound world he created.<br>P12: [...] for this music one has to have a lot of spirit with a lot of concentration, and you have to be there because this music demands everything from you [...] this music asks you to be totally naked and to jump off the precipice.<br>P13: [...] I had listened to works that were more deconstructive on the piano, I remember starting with those works by Cage in the 50s, on the prepared piano, and some works by Stockhausen that I had heard, but they were not so radical [...]
Action of listening	Control	Low	P1: Suddenly I focus on the rhythm, I listen today and I focus on the rhythm, and tomorrow I'm going to focus, I don't know, as I feel, I might listen to the timbre.<br>P2: [...] once that first listening was over [I frustratingly tried to repeat] the experience [...] Not only is it going to sound different on the speakers than in the hall, but also the interaction of my space and the speakers make certain things happen, then very soon I realized that this was what justified the repeated audition for me.<br>P8: I saw the large cymbal and the performer's hands on the screen and [...] I was suddenly surprised or interested [...] immediately, it came to my mind the piece by James Tenney [Having Never Written a Note for Percussion] and I said - wow! - […] I wouldn't know how to explain it because they were like all the thoughts that came to me at that moment [...]<br>P10: […] there is this moment where we hear short sounds, thumping sounds, like *tack* […] That was quite a strange sound, and I kind of still remember it [from the first listening].<br>P12: [...] I had no space to think, I was there immersed in the joy, in the revelry of all those sounds that arise, those structures that are so generous, one on top of the other -Wow!-<br>P13: [...] the percussions that he performs with the keyboard are very subtle and there are some timbres that on [the first listen] were confused with my space, because there are still some percussions that come from outside, suddenly something falls or the wood rattles, etc. [...]

		High	P1: That sound which enters there - tttttt- I do not wonder what instrument or what object is doing it, but [...] where is it that this thing [sound] is heading to?<br>P2: [...] one begins to hear many notes and even small melodic impulses that do not go beyond being mere impulses, or perhaps the melody is so poor, by its nature, that it falls more into the category of texture.<br>P3: [...] at first, nothing happens, it's just like the sound of the comb overlapping, but there is a moment where they begin to move from left to right.<br>P4: [...] the composer resorts to well defined rhythmic elements in the middle as a climax because they are totally different from the rest [of the piece] so I feel this is what captures my attention the best, it seems to me that it is a resource that she saves very well [until then].<br>P5: [...] in the end it's like a synthesis of the elements previously presented [...] that's why it's memorable, it's like [Berio] is preparing the listener for that moment to be remembered and appreciated.<br>P6: The first moment of tension comes, which is exactly where you paused the recording, where the other frequencies appear and where the spectrum opens up in a super interesting way. We have always been between the medium and low register, and now little by little things are going to start entering the register of the tweets.<br>P7: In the last movement there is a kind of coda [...] a timbral hybridisation of things that one has already heard before […]<br>P8: [The first time] I listened to it, I don't know, it must have been about five times from start to finish, and specific parts I think about seven times, that is, specific moments.<br>P9: [...] it is communicating an idea by resorting to more primitive elements than tonal ones, only sound organisation, that's what I mean by primitive, we can clearly perceive the rise and then the descent [on the flute], and with that I finish a musical idea.<br>P10: I could see, even the first time I heard it, something is rolling, something is moving. I wasn't sure about how exactly he was producing sounds […]but I could hear that there was something rolling […]<br>P11: [...] I chose the fourth piece because, of the first, third and fourth, I found that it was the best composed. For example, inserting those noises from a lock, those footstep noises [...]<br>P13: It is in this part that he already begins to mix the sounds that he has already presented, and that is where the central part of the work begins for me because all the timbres begin to combine.<br>P14: […] string gestures […] you see, line, line, line […] I suppose string players have to play in a way like hammering almost [[she moves her right arm as if playing repeated downbows on a violin]].<br>P15: [...] I start to draw [the musicians] according to when they play and then I leave them when I have to draw another section that is participating and then I return to them when the music returns to that side or when they are in tutti [...]
	Modality	Unimodal	P1: [...] because it's just beginning [...] on my first listen, I didn't feel like it was going to be a huge mass, perhaps they could have always stayed piano.<br>P2: The cluster was the least present [...] what was happening with the harmonics was absolutely amazing.<br>P3: [It sounds] more or less random, but with a certain density, a little at first, then a little more, and then a lot.<br>P4: [...] they were determined pitches, but since they were glissandi, they were all uncertain, so that seemed cool to me.<br>P5: Another important thing is the accents, that is, very diverse attacks, especially in the brass and the piccolo, which mark moments or allow the listener to hear key moments.

			P6: [...] in this movement of this work, a kind of harmonic support is heard [...] it is a pedal because it is down and supports what is going to happen, but with air, with noise, with a very interesting space.<br>P7: […] but let's say they are hybrid in a more segmented way, between the continuum of the first movement and the chords of the second [...] It is the most interesting section due to the timbral mixtures and the articulation between the different listening fragments of that part.<br>P9: [...] you have an initial idea that is clear, then an accumulation of things that can also be clearly perceived [...] any way that accumulation does not allow you to predict what comes after this crescendo [...] after the crescendo it tells you that another musical idea is coming and it tells you that with the timbre, that is, the harp.<br>P10: […] he moved from non-tone sounds to tone-based sounds. We hear now tones, before […] the spectrum of the sound was dense, and we couldn't hear tones.
		Cross-modal	P1: We are talking in a corporeal sense […] that - tttttttt - I imagined the performer who started doing it bending over.<br>P2: Listening to the piece many times is valuable because as I said, the final or the beginning will always be absolutely mediated by one's own experience and the space in which one is.<br>P3: Knowing where the sound comes from causes curiosity or interest […] I mean it is like an illusion [that] can only happen with a visual element, be it live or multimedia.<br>P4: [...] the flashes of high notes that stand out are very beautiful [...] it is very delicate so it does seem like very, very delicate streaks of light there, cool.<br>P6: […] you are not sure if the resonance is coming from the guitar or the computer; that's what I like.<br>P8: […] taking the stick near the tip and scratching it against the cymbal generates certain harmonics, then taking it a little higher or leaving a little more wood to scrape the cymbal generates other harmonics [...] is something I heard before seeing the score; I was noticing how he took the stick, how he moved it, how he scraped it and what sounds it generated when passing through the small grooves of the cymbal [surface ...]<br>P10: I remembered it [the thumping sound]. I did not know though how he made that sound. I mean, you know it is a piano but you don't know its causality. But then, when I saw it, and understood exactly how the sound is produced, that was more surprising.<br>P11: […] therefore the noises of running footsteps appear, which here break with that discourse, introducing an important dramatic element [...] I think that the choice of [sounds] of footsteps and shortness of breath speaks to us of a certain escape […]<br>P13: [...] well here we are talking about something that has more to do with culture, with the tradition of listening to the piano, I would still realise that it is a piano, but on the other hand it was not the interpretation of the piano, they are percussive effects that are performed on it [...]<br>P14: […] we don't really see it right now, because we are looking at the score, but imagine yourself you are in a concert hall, and you're seeing that. Then yeah, of course it's a really dramatic moment you will never forget.<br>P15: [...] I had heard the piece [live...] so they were two very different worlds. First, because of the musicians I saw [...] I draw sketches of all the concerts [I assist].
	Focus	Local	P1: I'm sure it was an oscillator [...] My mind after a certain time, it was seconds, said - here you can repeat this again -<br>P2: […] there is always a point, it can last more or less, depending on the version, where I don't know that what I am listening to is actually a gong.

			P3: Like [in the second] 45 or so, [the sound of the comb] starts to move from left to right [...] the playback is no longer one to one, it sounds like [a glissando] from low to high or high to low.<br>P4: […] because of the dynamics and [because of] some small glissandi that go up and down the same way, so I kind of feel that it makes the oscillation explicit and well, the flashes there of high notes that stand out are very nice.<br>P5: Around minute 13 I really liked the use of the [...] flute and the clarinet, which have a very soft timbre [...] Through these instruments, the tension that had been generated calms down a bit.<br>P6: Here where something that seems human begins to appear [...] that emanates from that pedal […] that moment in which the crescendo begins [...] comes the first moment of tension [...]<br>P7: [...] I am quite interested in the beginning of the piece [...] this perpetual continuum between the instruments and at the same time placing chords that have a super clear tonal connotation.<br>P8: [...] he passes the stick through the little grooves [of the cymbal] like this and suddenly he generates little taps [...] I also liked that part a lot.<br>P9: [...] there is a first idea that is an upward turn on the flute with the horns, then there is a downward turn on the same flute.<br>P10: […] the main theme is some sort of arpeggio, there is some sort of rolling sound, that I can see, even if I didn’t know what he was doing.<br>P13: [...] here he is already going somewhere else or he is introducing another timbre into the work.
		Global	P1: If [the piece] already presented me [with this climax from 3:00 to 6:00], my mind said: - you're going to do it again - but how are you going to present it? and that's when this happens [in 11:00], and that is a very different [new climax].<br>P2: I already said the extremes call my attention more, but I understand that I cannot have the extremes without the fortissimo [in the middle of the piece].<br>P4: [...] the composer resorts to well-defined rhythmic elements in the middle as a climax because they are totally different from the rest [of the piece] so I feel this is what captures my attention the best, it seems to me that it is a resource that she saves very well [until then].<br>P5: [...] in the end it's like a synthesis of the elements previously presented [...] that's why it's memorable, it's like [Berio] is preparing the listener for that moment to be remembered and appreciated.<br>P6: At this moment [...] the output of a deep processed voice that connects all [the characters] in a timbral gesture [...] is kind of climax [of the piece].<br>P7: In the last movement there is a kind of coda [...] a timbral hybridisation of things that one has already heard before, but let's say they are hybrid in a more segmented way, between the continuum of the first movement and the chords of the second [...]<br>P9: [...] and what happens when you reach the climatic point? It happens as it happens in a lot in the music from this period: a different element appears, the harp, and I think is followed by the xylophone, and then the trumpet, that is, timbres that did not appear before in the piece [...]<br>P10: […] the story here is he moved from non-tone sounds to tone-based sounds. We now hear tones, before […] the spectrum of the sound was dense and we couldn’t hear tones. […] that’s now, again, part of the journey he creates.<br>P11: The first part tells us about the beauty of this specific element that is the hinge […] but at the same time the

			composer knows that he cannot continue with that, and therefore the noises of running footsteps appear, which here break with that discourse, introducing an important dramatic element.<br>P12: [...] here one cannot look for the moment with contemporary music, what I liked, no, it is the complete work [...] there are moments, but as I told you, for me there are no reasons to remember those moments, it is about the totality of the piece [...]<br>P13: [...] the first material that he offers is essential to be able to later assess the intrusion into the harp and to capture well the use of the pedal or the tapping that he makes on the box [...]<br>P14: It begins with a musical saw crying, singing. That first gesture really grows into that big moment […] I like that climax moment because that was like the most exciting moment of the piece, and very, very active and intense, and before then we don't really hear the woodwinds and brass sections […]<br>P15: [...] I start to draw [the musicians] according to when they play and then I leave them when I have to draw another section that is participating and then I return to them when the music returns to that side or when they are in tutti, and I also realize that it is like a dislocation as they were not where I left them, they are at another time [...]
Mental mechanisms	Self-relevance appraisal	Idiosyncratic	P1: In my spare time I'm a poet, and so these pieces from *Aus den Sieben Tagen* sort of integrate poetry, music, alea, so that's what motivated me to choose it [for this interview].<br>P2: I quickly realised that what I like about that piece is not so much the act of listening to it but what happens to me after listening to it.<br>P3: I like the live versions better. Although it is not a piece that explores the space itself, in a live concert you hear how the sound becomes part of the space.<br>P4: [...] the first thing I thought was like wow! I want to see how they wrote that [...] I wanted to know how to get those timbres out of the instruments.<br>P5: [...] I like it better when [the pieces] are, like this one, on that edge [between the abstract and the programmatic].<br>P7: [...] When they ask me [...] what kind of contemporary music I appreciate the most, I always answer the music of my friends, and it's very true […]<br>P8: [...] the gestures when moving the sticks, scratching the cymbal and all that, of course it has everything to do with what is sounding, it is one of my favourite things.<br>P9: I don't think this music is very emotional in itself, I think few people listen to it, but I find it fascinating, I'm very excited that there are people who have done this in the context in which they did it.<br>P10: For me […] the gestures and the choreography come together with the sound. Then, [my interest is to know] what kind of contextual meaning, extra-musical meaning may have that thing that we hear.<br>P14: So, it is really, really important for me music has also something emotional or it creates some kind of visual element or expression, or it creates like magical moments I forget totally the time where I am, in the space where I am.<br>P15: [...] at the same time that I got to know the new music I began to be a tremendous fan of the new live music [...]
		Cultural	P2: [...] what matters most is the radicalism and discipline of the action. That is the first thing [the work] teaches; that's also why it's so cool that my first audition [of the piece] was live and I got to see the [disciplined actions of the] performer.

			P3: In this piece there are no elements that distract you from anything other than texture. [That was one reason for liking the piece]<br>P7: [...] that openness to those languages seems important to me, that non-hermeticism of Fagin's music seems important to me, it seems cool to talk about it.<br>P8: [...] that was also what I liked a lot. He explores a lot the sound of the cymbal [...]<br>P10: [In Guero] he really limited the sound palette to a very weird selection of sounds. In TemA there are a lot of beautiful moments, but they're moments, here and there. I wish he would've taken one moment and made a piece out of that […]<br>P13: [... in the times of the dictatorship] we clearly discovered that by listening to music from the market you run the risk of losing that freedom [...] contemporary music gave us that freedom because it did not allow itself to be tied down nor compromised with the elements that were in the daily life [...]<br>P15: [...] you could say Xenakis at 100 years old is already a classic, and it is a classic, but what happens when you listen to his work over and over again? That his ideas are truly fresh, striking, different from anything that has been done for many centuries.
	Attention	Covert	
		Overt/Analytic	P1: I'm sure it was an oscillator [...] My mind after a certain time, it was seconds, said - here you can repeat this again - and it didn't happen, something else happened. So, this kind of thing [...] causes me interest because it's like I'm being deceived.<br>P2: The less timbral perceptual clarity, the greater the manifestation of the harmonic series. [...] One begins to hear many notes and even small melodic impulses.<br>P3: [...] all you hear are the teeth of the comb, and suddenly you hear these pebbles [...] which is a granulator that captures the sound and converts it into pebbles.<br>P4: I find it very nice that many harmonics stand out in the middle of everything and the gesticulation of oscillations in this part is very explicit.<br>P5: [In this part] he maintains the intensity by various means, the brass and the woodwinds at their maximum power; I think he uses this technique in the third movement of the symphony.<br>P6: Here where something that seems human begins to appear [...] that emanates from that pedal [...] that moment in which the crescendo begins [...] comes the first moment of tension [...]<br>P7: [...] is that moment when there are these kinds of articulated flashbacks of complex elements that make up many parts of the piece [...] a moment of elements that are discontinuous but that work well in their discontinuity.<br>P8: […] I was really struck by that piece, how taking the stick near the tip and scratching it against the cymbal generates certain harmonics, then taking it a little higher or leaving a little more wood to scrape the cymbal generates other harmonics [...]<br>P9: [...] there is a first idea that is an upward turn on the flute with the horns, then there is a downward turn on the same flute. That itself is already a musical idea, which is built with primary elements, a melodic contour [...]<br>P10: Silence, right? We heard something pretty significant here, and he put that silence to reflect upon it; how great isn't it?<br>P11: [...] the change was important because that wall [of electronic sounds...] that great mass prepares us for this [...] footsteps and shortness of breath [that] speaks to us of a certain escape [...].<br>P13: [...] that is what caught my attention later, in the sense that here is a proposal, he does not want to offer you

			an experience of a certain timbre but rather it is a whole conjunction of elements that are being built and want to tell you something [...]
	Association	Indexical	P2: The timbral property of the instrument is not understood until the crescendo becomes more evident.<br>P3: [...] it seems very physical to me, it seems very sensory to me, as if the little pebbles were felt, or like little balls of sound... [[she makes a gesture with the tips of her fingers as if she had grabbed little pieces of something]].<br>P4: I wanted to know how to get those timbres out of the instruments […] what a blow how they come to sound so different from the solo instrument.<br>P5: Around minute 13 I really liked the use of the [...] flute and clarinet, which have a very soft timbre [...]<br>P6: Here where something that seems human begins to appear [...]<br>P8: [...] he passes the stick through the little grooves [of the cymbal] like this and suddenly he generates little taps.<br>P9: [...] you perceive an accumulation that goes hand in hand with a crescendo [...] the accumulation corresponds to a greater density, these two factors go hand in hand, therefore, it is not difficult to understand it.<br>P10: I could hear that there was something rolling, and it was rough, somehow, it had some sort of regular edges.<br>P11: [...] I think that the choice of [sounds] of footsteps and shortness of breath [...]<br>P13: [...] these timbres that he accumulates [...] show a kind of materiality of this instrument [...] The fingernail that slides, I'm not sure if it's the fingernail and the fingertip, that is very subtle, you hear it [...]<br>P14: I suppose string players have to play in a way like hammering almost [[she moves her right arm as if playing repeated notes on a violin]].
		Iconic	P1: here he is showing me a super high pitch in a forte [...] It is causing me this: super sharp sensation [while singing a high pitch he bangs his open left hand with the clenched fist of his right hand].<br>P3: [...] for me these small sounds are like [...] that magical feeling when you see fireflies that appear and disappear at different depths.<br>P4: That's where I feel that it sounds like a Doppler effect [...] I think that sound of sirens works very well there [...] it's like there were many mermaids riding waves, I find that amusing.<br>P5: [...] in the peak areas there is a greater density, a greater activation, and in the areas that are not like this there is relaxation, then, this relaxation allows us to perceive a state of mind of calmer thoughts.<br>P6: [...] is heard as that kind of harmonic support with sounds that are almost metallic, like wind, but that are not necessarily in tune [...]<br>P8: [...] that passage, apart from associating it [to the sound] of the bow when [rubbing] the cymbal, those squeaks [...] I associate them [...] with animals that do not exist [...]<br>P9: [Ligeti's] processes are more natural [...] you can equate them with a natural physical process, for example "Lux Aeterna" which is basically an elastic canon [where] each voice makes the same melodic contour, but each voice advances at its own pace, like when you add a drop of iodine into water. All the iodine particles make similar movements or a cloud, that is, they have the same tendency of movement with a certain interdependence.<br>P10: So, what we've just heard maybe it sounds to me like a kid is rolling a stick on a fence while he's walking, or you're running the wheel of your bicycle while you put in the spokes a little piece of wood or paper […]<br>P11: Picasso, in the period of synthetic cubism, glued things from outside to a piece of newspaper throughout the painting, that is, he takes an external referent and organizes a discourse on that. Here it seems the same to me,

			that's why I really like this composition [...]<br>P12: [...] everything is very steely, there is nothing in this music that is beautiful [...] Steely, just as if one passed sandpaper over an iron, I call that steely [...]<br>P13: [...] the first time I heard it [...] the sounds of a clock came to me, also of percussion instruments [...]<br>P14: With the musical saw he makes that crying kind of sound. Female crying.
		Conceptual	P1: […] it is what Stockhausen says metaphysically: 'all space'.<br>P2: What one sees is how the piece is swallowing the noise of the world and then falls back into it.<br>P3: [...] I find it incredible that a sound can be [break through a granulator]. In other words, something that is not very tangible, you can do that kind of process that is tangible.<br>P4: It's very dramatic, those melody lines at the end are very expressive, yeah, it's like they've already been beaten at whatever they're up there fighting for.<br>P5: I think the restlessness [I feel] comes from a very extreme feeling of emptiness and loneliness [...] total emptiness of things [...] a situation where I can't find anything that normally exists [...]<br>P6: [...] until this moment arrives where the voice is very evident, again we do not return to reality. It was clearly the connection with the fantastic [...]<br>P7: […] it is a moment of high discontinuity, but a moment of elements that are discontinuous but that work well in their discontinuity.<br>P9: [...] what most links me to Lachenmann is his political position [...] I like his music, but I find more value in it to the extent that I know more about his political position towards music.<br>P10: So, certainly I think even unconsciously these sounds are so primordial, somehow. And kind of resonate with you very deeply. They're not just abstractions but they are part of you, part of your life.<br>P11: [...] emotionally it speaks to us of a certain fragility, of a certain instability in the daily things in which we live, and this type of piece personally shows me that.<br>P12: [...] I want to say that contemporary music has suffering, all it has is what is currently happening with society, that is my conclusion.<br>P13: [...] the instrument that is here is the piano, but not in this case, and that's why I liked it, because in the end it doesn't want to make you recognise the piano because the experience itself is a kind of [...] reconstruction of it from [...] all these series of percussions that he performs.<br>P15: [...] Thalleïn, which means sprouts, and that at that time I did not understand why different very dense musical events suddenly happened and others that were not so dense, and those silences [...] but that his name gave me clues [...]
	Musical expectancy	Unimodal	P1: I'm sure it was an oscillator [...] My mind after a certain time, it was seconds, said - here you can repeat this again - and it didn't happen, something else happened. So, this kind of thing [...] causes me interest because it's like I'm being deceived.<br>P3: [It sounds] more or less random, but with a certain density, a little at first, then a little more, and then a lot.<br>P4: Well, since [the work] is so progressive, I think the expectation is that it grows to the top and then goes down again, but [the rhythmic part of the middle of the piece] is like an ace up the sleeve, very well used.<br>P5: The accents also help to emphasize a call for attention [...] an unexpected event draws attention, but I don't really know what it is, it could be violent, it could be non-violent, it could be distressing, not distressing, that really I don't know, however, a sudden event does lead me to focus my attention towards that place.

			P6: [..] it stops the possibility of receiving confirmation of what you hear from what you see […]<br>P7: [...] There is a moment, around the second 30th, in which he makes some major chords than one cannot expect at all [...] they are well tonal chords.<br>P9: [...] I think it would be very difficult for someone hearing this for the first time and stopping the recording right at the crescendo to believe that the harp is going to follow [...] But if you stop the recording two bars before the crescendo a lot of people would tells you that a crescendo is going to follow because […] the sum of the factors that are there in the piece tell you that it will follow a crescendo in a little while.<br>P10: [The harp stroke] is a good surprise, and it came at the right moment, like when you start feeling you've heard that. So, is this all about this piece? And then it comes that new element […] and then it feels yeah, he's on it, it continuous.<br>P12: [...] all those basses are a whirlwind, it is a whirlwind that prepares what is going to come after, which is a wall of sounds [...] that is the orchestra that is developing.<br>P13: A hit he makes on the box, well, that sound was undoubtedly a warning call. Regardless of whether it is different, the fact that he intends to do something with his discourse, that he intends to incorporate us into a new dimension, it is a call or a closure, I don't know; one is always building the work in that aspect.<br>P14: I like that climax moment because that was like the most exciting moment of the piece, and very, very active and intense, and before then we don't really hear the woodwinds and brass sections […] how effective that was, the entry of the brass section especially.
		Cross-modal	P1: In that part, the qualities of the saxophone are heard a lot. So, voluntarily or involuntarily, sometimes images of free jazz performance come to mind, and I don't like that […] if it's not doing effects, [the saxophone] tends to [...] remind you of traditional music [...]<br>P2: It never ceases to amaze me, regardless of whether I already know what the work is like, [...] that there is always a point, it can last more or less, depending on the version, where I don't know that what I am listening is actually a gong.<br>P3: Knowing where the sound comes from causes curiosity or interest […] I mean it is like an illusion [that] can only happen with a visual element, be it live or multimedia.<br>P4: I wanted to know how to get those timbres out of the instruments […] what a blow how they come to sound so different from the solo instrument.<br>P6: […] it stops the possibility of receiving confirmation of what you hear from what you see […] you hear the guitar being played in the centre [of the "real space"] and the [processed guitar sounds] go around the public, as if the guitar were embracing it.<br>P8: [...] the surprise itself was due to seeing the person with the sticks and his hands placed [on the cymbal...] and remembering the piece by James Tenney, at that moment I began to listen to it [...] and at the first strike of [the percussionist] I said: - Wow! this is going to be good -<br>P10: I guess it's quite strong, that besides hearing the sounds you […] also hear the gesture of the sound, even if you don't know the source, you still hear that things are rolling left to right.<br>P11: [...] that same use of contrast of violent, harsh things, relaxing sounds, like atmospheric and suddenly [...] emerge as a wall of strong electronic sounds that contrast and prepare for what is to come [...] the noises of running footsteps appear, which here break with that discourse, introducing an important dramatic element [...] I think that the choice of [sounds] of footsteps and shortness of breath speaks to us of a certain escape […]

			P13: [...] it seems to me that he is building a new instrument [...] in fact this is what takes you there because of course the piano is distorted, it is undoubtedly the base material [...] he is working on a dialogue between the virtual instrument [the güiro] on the piano, but it is neither one nor the other [...] after tapping on the box, the actual construction of this new instrument begins.<br>P14: It begins with a musical saw crying, singing. That first gesture really grows into that big moment […] Listening to that piece in a concert hall […] you know we don't see a hammer in a concert hall normally, a percussionist playing hammer, right? so when you see that force, when you see that sound, we see it and we hear it, so it becomes like double experience.
Affects	Locus	Felt	P1: In this part it makes me laugh and at the same time joy, it's like being super euphoric in this mass.<br>P2: [The piece is] a genuine invitation to be there [...] from an emotional point of view it is undoubtedly very liberating, I think, to be able to give up the fight against reason for a while.<br>P3: Knowing where the sound comes from causes curiosity or interest […] I mean it is like an illusion [that] can only happen with a visual element, be it live or multimedia.<br>P4: I think I remember that [the first time] I heard it, at one point it filled up, the texture thickened a lot and suddenly I was a little overwhelmed.<br>P5: The more I listen to the piece the delight becomes somewhat technical.<br>P6: […] you are not sure if the resonance is coming from the guitar or the computer; that's what I like.<br>P7: [...] within these textures, heterogeneous elements can be combined to create slightly surprising textures [...] that's the word, surprising.<br>P8: [...] at the end I said: - how on earth? - I was even more interested […]<br>P9: [...] I feel more emotion in the link with the period of Webern and Schönberg because they underwent a radical change of language, on the contrary, Sciarrino and Xenakis did not have to make that radical change, they had to find different ways of making music, once the change has been made.<br>P10: […] that sound energy that you can identify […] that engages you more, it makes you more related to this piece, and more connected to this piece.<br>P11: What I liked about the whole record was the use of language. The guy has a good command of what he is talking about, how he mixes, the effects he uses, how he intersperses them [...]<br>P12: [...] as it is music with a lot of uncertainty, my life is uncertain, the music portrays me, I feel portrayed. [...] And that frees me, it’s like feeling well.<br>P13: [...] also being able to predict other possibilities, other timbres, other instances that may be configured in the piece, that is the freedom that I believe is given here; we could define that as affective freedom that is being worked on here.<br>P14: I think the most important thing is, it makes me forget about what time is right now, and what I have to do, I totally forget about, I don't care because it's so gorgeous, so beautiful […]<br>P15: [...] my passion for this music is that I don't know what is really going to happen and it gives me great pleasure to know that we are all in the same circumstances, at least the public [...]
		Recognised	P5: [...] in the peak areas there is a greater density, a greater activation, and in the areas that are not like this there is relaxation, then, this relaxation allows us to perceive a state of mind of calmer thoughts.<br>P9: [...] I enjoy that elegance [of Sciarrino’s timbral design], but I look at it more from the outside, that is, I’m not so emotionally involved.

			P11: [...] that same use of contrast of violent, harsh things, relaxing sounds, like atmospheric and suddenly [...]<br>P12: [...] the music with the text seems to me an exhortation, it has tremendous power, a tremendous force that unfolds until it dies down, but it does not melt into some melancholy [...]<br>P13: That conjugation of all the timbres, and it's like a time for reflection too, it's not something very hectic or anything like that, it's a work that gives you space [...]<br>P14: There is some kind of tragedy in this piece, rather than sadness.
	Valence	Negative	P1: in that part the qualities of the saxophone are [timbrically] obvious, […] I don't like that.<br>P2: [A couple of times] I didn't adjust the volume well before sitting down to listen, then of course [during the fortissimo] there came the point that was […] annoying.<br>P4: [During the first listening experiences] there were parts where the sounds evoked more negative feelings; They caused me a little anguish, but I still really liked the overall experience.<br>P5: I think the restlessness [I feel] comes from a very extreme feeling of emptiness and loneliness<br>P9: [...] Xenakis's speech is very difficult, it's very rough for me [...] it's hard for me to understand how he's going to carry it out, at least if I were the composer [...]<br>P10: I wish he would've taken that moment an made a piece out of that, but he doesn't do it in TemA. So, for me that kind of throws me away.<br>P11: [...] experimental music, electroacoustic and serial music are not happy, so essentially the themes tend to be sort of dramatic.<br>P12: [...] this music by Nono, for example [...] the incessant noise, something that does not stop, the fracture, the rage, the impotence [...]<br>P14: Pithoprakta […] during the class you talk about it, but I don't love it […] it doesn't touch me, it doesn't speak to me […]it sounds cold to me […]
		Positive	P1: It gives me satisfaction, I say - how beautiful - how did he come up with that noise there? Right there.<br>P2: I would define [those feelings] as fascination for the beginning and the end of the piece.<br>P3: With good headphones, I think it kind of isolates you and takes you to another world […] it continues to be interesting and pleasant.<br>P4: I feel like this is the part that's most obvious that it's a prolonged texture that gives a lot of peace [...] it's at the same time an ebb and flow of dynamics, but overall it's been pretty stable.<br>P5: [...] it is the material with which he begins in a fairly calmed timbral situation.<br>P6: He has a very broad portfolio of climaxing strategies [...] this is one; where not necessarily with dynamic growth, nor with spatial movement, there is something almost like a harmonic cadence to solve, and I like that.<br>P7: The feeling of beauty is essentially linked to the feeling that there is an explicit imagination effort […]<br>P8: [...] apart from that [initial] surprise that in fact was maintained from beginning to end, I was expecting everything that the interpreter was doing at all times; It was like an explosion in my head of a lot of ideas that I could do [...]<br>P9: […] I find it fascinating, I'm very excited that there are people who have done this in the context in which they did it.<br>P10: That's why I say I can resonate with his train of thought, with his music. That's what made that experience profound and memorable […]<br>P11: […] apart from showing the beauty of that specific sound [...] inserting those noises from a lock, those

			footstep noises, mixing them, because he processes them, then he [presents us with] well-built electronics [...]<br>P12: [...] I had no space to think, I was there immersed in the joy, in the revelry of all those sounds that arise, those structures that are so generous, one on top of the other -Wow!-<br>P13: [...] I liked it, I found it very direct, very clear, very precise in the first audition.<br>P14: Penderecki's music has here and there passion, excitement, sadness [..] it has some kind of deep feeling. So that's what I really like about this piece.<br>P15: [...] my passion for this music is that I don't know what is really going to happen and it gives me great pleasure to know that we are all in the same circumstances, at least the public [...]
	Specificity	Non-specific	P1: At first [I feel] a little surprised or interested in knowing what is happening or what is going to happen, what's next.<br>P2: I would define [those feelings] as a fascination for the beginning and the end of the piece.<br>P3: Knowing where the sound comes from causes curiosity or interest […]<br>P4: I chose it because it was a pleasant surprise when I heard it for the first time because it is a very unexpected way of reaching something very nice.<br>P5: [...] the emotion is no longer directly associated with something personal, but it becomes a kind of recognition of an emotional area but not directly linked to something that is biographical.<br>P6: That whole part is very beautiful because it is clearly like a kind of incredible spectral resonance of the voices.<br>P7: The feeling of beauty is essentially linked to the feeling that there is an explicit imagination effort by the composer to look for a particular combination and make it emerge in a magical way in a context in which one cannot expect it.<br>P8: [...] at first, it was the surprise to see the performer there with the sticks and the cymbal [...]<br>P9: […] I find it fascinating, I'm very excited that there are people who have done this in the context in which they did it.<br>P10: […] that sound energy that you can identify […] that engages you more, it makes you more related to this piece, and more connected to this piece.<br>P11: The first part tells us about the beauty of this specific element that is the hinge [then …] the noises of running footsteps appear, which here break with that discourse, introducing an important dramatic element.<br>P12: [...] my life itself, with all its fears, with everything that I may be hiding right now and with all that you may be knowing me, that is what I feel about contemporary music.<br>P13: [...] my listening from the first to the last, which I think was yesterday, was very, very good, it was always different, always interesting, that's why I've chosen it.<br>P14: So that's what I really like about this piece. It spoke to me; it touched my heart.<br>P15: [...] my passion for this music is that I don't know what is really going to happen and it gives me great pleasure to know that we are all in the same circumstances, at least the public [...]
		Specific	P1: In the section where I told you that there is only the saxophone and the piano, I also feel that way [...] I feel peace.<br>P4: [...] the way they play with those sounds creates a landscape that I find interesting and meditative, and that meditation is cool because it makes me feel at peace there.<br>P5: I began to think about the effects of calm, intensity and drama [...] the entire initial part is very calm.

			P14: Penderecki’s music has here and there passion, excitement, sadness […]
Concepts	Dynamism	Single	P1: It is what Stockhausen says metaphysically: 'all space' [...] In that passage [where texture is reduced] I feel that immensity that is not heard.<br>P3: [...] in the live concert you feel the panning better because there is a lot of panning of some sounds that move from one side to the other.<br>P5: I think the restlessness [I feel] comes from a very extreme feeling of emptiness and loneliness [...] total emptiness of things [...] a situation where I can't find anything that normally exists [...]<br>P6: [... Dhomont] allows you to say: - From what part of the real world do these things come to the magical world?<br>P7: […] It is a moment of high discontinuity [of elements that …] in their heterogeneity work quite well, and there is surprise [in the transition from] one to the other […]<br>P8: [...] I am sure that the perception of the piece would have been totally different if it had not been miked in that way [...]<br>P9: [...] the accumulation corresponds to a greater density, these two factors go hand in hand, therefore, it is not difficult to understand it.<br>P10: the gestures and the choreography come together with the sound.<br>P12: [Contemporary music] portrays me, frees me.<br>P13: [...] the sounds that one hears resist an interpretation that could be given regarding a social situation, a tradition, and in that aspect they remain very pure, very authentic [...]<br>P15: [...] We listen with our whole body, we are vibration, so we also participate in these different vibrations, I feel that this is captured a lot in live experiences, in that rite, in that live ritual [...]
		Narrative	P1: And I feel this part as a kind of abandonment of leaving all this mass [...] we cut the tie that kept us in the mass that happened before.<br>P2: What one sees is how the piece is swallowing the noise of the world and then falls back into it.<br>P4: [...] it's like a lot of tension generated [...] and as if everything ceased out of nowhere and nothing mattered, and it just loosens up and ends without going further and that's the final dialogue of defeat.<br>P6: [...] until this moment arrives where the voice is very evident again, we do not return to reality. It was clearly the connection with the fantastic [that happened right before this last section].<br>P11: [...] that same use of contrast of violent, harsh things, relaxing sounds, like atmospheric and suddenly [...] emerge as a wall of strong electronic sounds that contrast and prepare for what is to come [...] the noises of running footsteps appear, which here break with that discourse, introducing an important dramatic element [...] I think that the choice of [sounds] of footsteps and shortness of breath speaks to us of a certain escape […]<br>P13: [...] he chose that part of the harp, and that introduces subtlety [...] And it gives his work a lot of depth there because in the same way that he gets more [physically] into the piano, his work begins to acquire timbres that are quite delicate.
